# Obesity promotes radioresistance through SERPINE1-mediated aggressiveness and DNA repair of triple-negative breast cancer

**DOI:** 10.1038/s41419-023-05576-8

**Published:** 2023-01-21

**Authors:** Yong-Han Su, Yi-Zhen Wu, David K. Ann, Jenny Ling-Yu Chen, Ching-Ying Kuo

**Affiliations:** 1 Department of Clinical Laboratory Sciences and Medical Biotechnology, College of Medicine, National Taiwan University, Taipei, Taiwan; 2 Department of Diabetes Complications & Metabolism, City of Hope, Duarte, CA USA; 3 Irell and Manella Graduate School of Biological Sciences, City of Hope, Duarte, CA USA; 4 Department of Radiology, National Taiwan University College of Medicine, Taipei, Taiwan; 5 Department of Radiation Oncology, National Taiwan University Cancer Center, Taipei, Taiwan; 6 Department of Laboratory Medicine, National Taiwan University Hospital, Taipei, Taiwan

**Keywords:** Breast cancer, Double-strand DNA breaks, Radiotherapy, Obesity

## Abstract

Obesity is a risk factor in various types of cancer, including breast cancer. The disturbance of adipose tissue in obesity highly correlates with cancer progression and resistance to standard treatments such as chemo- and radio-therapies. In this study, in a syngeneic mouse model of triple-negative breast cancer (TNBC), diet-induced obesity (DIO) not only promoted tumor growth, but also reduced tumor response to radiotherapy. Serpine1 (Pai-1) was elevated in the circulation of obese mice and was enriched within tumor microenvironment. In vitro co-culture of human white adipocytes-conditioned medium (hAd-CM) with TNBC cells potentiated the aggressive phenotypes and radioresistance of TNBC cells. Moreover, inhibition of both cancer cell autonomous and non-autonomous SERPINE1 by either genetic or pharmacological strategy markedly dampened the aggressive phenotypes and radioresistance of TNBC cells. Mechanistically, we uncovered a previously unrecognized role of SERPINE1 in DNA damage response. Ionizing radiation-induced DNA double-strand breaks (DSBs) increased the expression of SERPINE1 in cancer cells in an ATM/ATR-dependent manner, and promoted nuclear localization of SERPINE1 to facilitate DSB repair. By analyzing public clinical datasets, higher *SERPINE1* expression in TNBC correlated with patients’ BMI as well as poor outcomes. Elevated SERPINE1 expression and nuclear localization were also observed in radioresistant breast cancer cells. Collectively, we reveal a link between obesity and radioresistance in TNBC and identify SERPINE1 to be a crucial factor mediating obesity-associated tumor radioresistance.

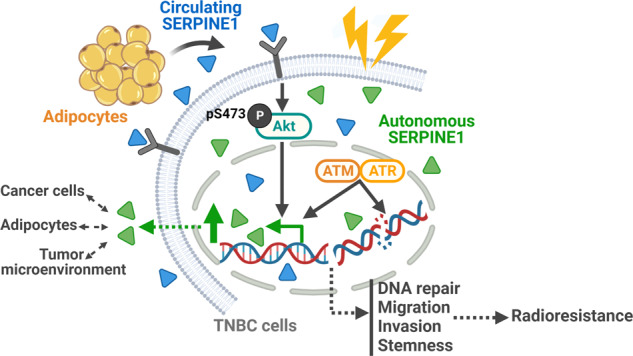

## Introduction

Obesity has been a growing issue worldwide. It has been recognized as a risk factor for various metabolic disorders, including cardiovascular disease, type 2 diabetes, and cancer, including breast cancer. Patients who are overweight and obese exhibit higher tumor recurrence rate and metastatic potential than those who have normal weight, irrespective of their menopausal status [1–3]. Moreover, obesity has been associated with poor outcomes of cancer treatments, including chemotherapy and radiotherapy (RT) [4–8]. Despite epidemiological evidence links obesity to increased cancer risk and poor prognosis, the underlying mechanism remains elusive.

RT is the first-line treatment for cancer patients either alone or in conjunction with chemotherapy or surgery. More than half of cancer patients receive curative or palliative RT, but the response varies among individuals. While ionizing radiation (IR) effectively treats many types of tumor initially, radioresistance often leads to treatment failure and causes tumor recurrence or metastasis. The development of resistance to RT can be complex and involves many factors and mechanisms, including deregulated DNA damage response (DDR) signaling, enhancement of epithelial-mesenchymal transition (EMT), stemness potential, and metabolic reprogramming [9–12].

Triple-negative breast cancer (TNBC) accounts for 15–20% of invasive breast cancer worldwide and exhibits heterogeneous molecular features [13, 14]. According to epidemiologic studies, incidence of TNBC vary with race and ethnicity, possibly owing to differences in lifestyle, including both biologic and non-biologic factors [15, 16]. Due to the lack of therapeutic targets (ER, PR, and HER2), RT and systemic chemotherapy are often used pre- or post-operatively for treating TNBC [17]. Although TNBC cells are deficient of hormone receptors, and the obesity-mediated hormonal mechanisms have little impact on TNBC progression, adiposity is still considered as a malignant factor for TNBC. The obese state has been linked to higher incidence, progression, and therapeutic resistance in TNBC [18–20]. However, the relationship between obesity and tumor radioresistance is still unclear.

SERPINE1(serpin family E member 1, also known as plasminogen activator inhibitor 1, PAI-1) was first identified as an inhibitor of the plasminogen activator system to regulate fibrinolysis and proteolysis. It is produced by various cell types such as endothelial cells, fibroblasts, smooth muscle cells, and adipocytes [21–23]. SERPINE1 is also found to closely correlate with pathophysiological conditions including obesity and metabolic syndrome such as cardiovascular diseases, insulin resistance, and type 2 diabetes [24, 25]. In addition, accumulating evidence shows that SERPINE1 promotes cancer progression, metabolic alteration, metastasis, and even therapeutic resistance in several types of cancer, including breast cancer [21, 26–31]. Since the breast is largely composed of adipose tissue, SERPINE1 has been shown to correlate with obese status and cancer progression in breast cancer patients and may be a prognostic biomarker and therapeutic target [31, 32]. However, whether SERPINE1 links obesity to breast cancer progression or therapy response remains elusive.

In the present study, we set out to investigate the impact of obesity on the response to RT in TNBC. We report that diet-induced obesity (DIO) promoted radioresistance in a syngeneic mouse model of TNBC. In addition, culturing human TNBC cells with human adipocyte-conditioned medium recapitulated our observation in vivo. TNBC cells acquired aggressive phenotypes and resistance to IR after exposure to adipocyte-secreted factors. Importantly, we found that SERPINE1 was enriched in tumor cells and the surrounding microenvironment in response to DIO and IR. Both circulating SERPINE1 and intracellular SERPINE1 play key roles in driving radioresistance of TNBC under obese or obesity-mimicking conditions. Moreover, SERPINE1 not only promoted cancer aggressive phenotypes, but also elicited a non-canonical function in facilitating DNA double-strand break (DSB) repair in TNBC cells, thereby resulting in resistance to IR. Pharmacological inhibition of SERPINE1 by a small molecule compound, tiplaxtinin, resensitized tumors to IR in DIO mice. Our study reveals that obesity leads to poor response to RT and pinpoints SERPINE1 as a critical regulator, shedding lights on the development of prognostic markers and therapeutic targets for TNBC patients undergoing RT.

## Materials and methods

### Diet-induced obesity and syngeneic mouse model of TNBC

Animal experiments were approved by the Institutional Animal Care and Use Committee at College of Medicine, National Taiwan University. 4-week-old female C57BL/6 mice were randomly separated into two groups and fed with control diet (CD, 10% kcal from fat) (Research Diets, D12450J, New Brunswick, NJ, USA) or high fat diet (HFD, 60% kcal from fat) (Research Diets, D12492). 4 weeks after starting CD or HFD, mice in the CD or HFD group were selected for cancer cell implantation, based on the criteria for body weight and fasting glucose level (CD: body weight: 18.92 ± 1.96 g; fasting glucose: 69.08 ± 25.62 mg/dL. HFD: body weight: 21.82 ± 4.30 g; fasting glucose: 100.86 ± 35.85 mg/dL). Criteria were determined empirically. 2.5 × 10^5^ EO771 cells were injected into each mouse. Next, mice in CD or HFD group were then further randomized into two groups (NO IR or IR), and fractionated radiation (40 Gy/4 fx or 40 Gy/5 fx in tiplaxtinin test) was performed when the tumor approximately reached 50 mm^3^ (tumor volume = length × width^2^/2). Subcutaneous injection of cancer cells into the right hindlimb instead of orthotopic implantation in the mammary fat pad was performed to avoid the radical particles damaging the central organs during IR. For the tiplaxtinin test, mice in CD or HFD group were all undergone IR. Tiplaxtinin was dissolved in corn oil (Sigma, C8267, Saint Louis, MO, USA) with 10% DMSO and supplied by oral gavage daily for 14 days. Body weight, food intake, and tumor volume were measured every 3 days till the endpoint of experiments. Mice were euthanized when the largest tumors reached ~1000 mm^3^, the size that might impede the movement of mice. Tumors and the inguinal white fat pad (iWAT) were excised and weighed after euthanasia. For measuring biochemical markers, mice were fasted overnight before submandibular blood collection. Fasting glucose was assayed using whole blood by a blood glucose meter (HMD Biomedical, GlucoLeader Enhance, Hsinchu, Taiwan.), and serum fasting insulin (Cloud-Clone, KSA448Mu11, Katy, TX, USA) and Serpine1 (R&D System, DY3828, Minneapolis, MN, USA) were measured by ELISA.

### Cell culture and reagents

MDA-MB-231, MDA-MB-436, HS578T, BT20, MDA-MB-468, MCF7, and HEK293FT cell lines were cultured in DMEM (Gibco, 12100046, Waltham, MA, USA). T47D, BT549, and BT474 cell lines were cultured in RPMI 1640 (Gibco, 31800022). All cell lines were obtained from ATCC (American Type Culture Collection, VA, USA) or BCRC (Bioresource Collection and Research Center, Hsinchu, Taiwan), and maintained at 37 °C with 5% CO_2._ Cell culture medium was supplemented with 10% FBS (Gibco, 10437028), 3.7 g/L sodium bicarbonate (Sigma, S5761, Saint Louis, MO, USA), 1 mM sodium pyruvate (Gibco, 11360070), 100 U/mL Penicillin, 100 μg/mL Streptomycin and 250 ng/mL Amphotericin B (Gibco, 15240062). 0.023 U/mL of insulin was added for maintaining BT549. Radioresistant MDA-MB-231 cell line was established as previously described [33]. All cell cultures were periodically tested for mycoplasma. Tiplaxtinin (9000450), MK2206 (11593), VE821 (17587), KU55933 (16336) and doxorubicin (15007) were from Cayman Chemical (Ann Arbor, MI, USA). Recombinant human SERPINE1 (140-04) was from PeproTech (Cranbury, NJ, USA), and recombinant human insulin (12585014) was from Gibco.

### Human white adipocyte differentiation and collection of conditioned medium

Human white preadipocytes (hPAd) derived from female abdominal subcutaneous adipose tissue of at least two different individuals were obtained from PromoCell (C-12730, Heidelberg, Germany). hPAd were cultivated in growth medium (PromoCell, C-27410), followed by switching to differentiation medium (PromoCell, C-27436) for 3 days, and then matured in nutrition medium (PromoCell, C-27438) for additional 15 days to become mature human white adipocytes (hAd). hAd-conditioned medium (hAd-CM) was collected every 3 days until day 45. Cellular debris was removed by centrifugation, and the supernatant was stored at −80 °C. Several collections of hAd-CM were pooled as one batch of hAd-CM and SERPINE1 concentration was measured by ELISA before use. The average SERPINE1 concentration was 16.44 ± 2.81 ng/ml in different batches of hAd-CM. For SERPINE1-depleted hAd-CM, hAd were transduced with lentivirus containing shScramble or shSERPINE1 24 h after maturation. For all the experiments, cancer cells were pre-treated with hAd-CM mixed with DMEM at 1:1 ratio for 3 days and were maintained in the same media throughout the experiments. Mature hAd culture medium (nutrition medium) mixed with DMEM (1:1) was used as a control.

### Ionizing radiation

Ionizing radiation was administered using an IBL-637 cesium irradiator (Cs-137, 270 cGy/min dose rate, Gamma Service Medical GmbH, Leipzig, Germany). For mouse tumor studies, a custom-made lead shield was used to deliver RT to tumor and minimal dose to other areas.

### Transwell co-culture assay

Co-culture assay was performed using a Transwell system (0.4 μm pore size, PET membrane) (Corning, CIR3470, Corning, NY, USA). MDA-MB-231 cells were plated in the inserts at a density of 1000 cells per well, and inserts were transferred into hAd-containing chambers for 3 days before IR. Cancer cells were exposed to IR alone, and the cell viability was further evaluated by ACP assay 3 days post IR.

### Acid phosphatase (ACP) assay

For cell viability analysis, cells were seeded in a 96-well plate and exposed to indicated dosage of IR. After incubation of 96 h (for MDA-MB-468 cells) or 168 h (for BT20 cells), 7 mM of para-nitrophenyl phosphate (Sigma, N4645) in acid phosphatase buffer (0.1 M sodium acetate with 0.1% Triton X-100, adjusted pH to 5) was supplied to each well and incubated at 37 °C for 40 min. Then, 1 N NaOH was added to lyse cells, and the absorbance was measured at 410 nm using SpectraMax Plus 384 (Molecular Devices, San Jose, CA, USA). For IC_50_ analysis, cells were incubated with the indicated concentration of inhibitor for 72 h, and the harvesting processes were as described above.

### Clonogenic survival assay

Single cells were plated and irradiated at 2, 4, 6, and 8 Gy on the next day. After 7–10 days, cells were fixed with ice-cold methanol and stained with 0.2% crystal violet. Colonies were enumerated and surviving fraction was calculated with plating efficiency.

### Transwell migration/invasion assay

Cell migration and invasion were assessed by Transwell system (8 μm pore size, PC membrane) (Corning, COR3422). For Matrigel invasion assay, the transwell insert was pre-coated with 1:8 diluted Matrigel (BD, 356231, Franklin Lakes, NJ, USA). Cells were resuspended with serum-free DMEM and plated in the insert at a density of 1 × 10^4^ cells per well and the lower chamber contained the indicated culture medium. After 24 h of incubation, cells were fixed with ice-cold methanol and stained with 0.2% of crystal violet.

### Tumorsphere formation

Tumorsphere culture was performed as previously described [34]. For serial passaging, tumorspheres were collected, dissociated, and reseeded at a density of 5000/2500 cells per well for the secondary/tertiary culture, respectively. Tumorspheres with diameter >200 μm were enumerated.

### RNA extraction, cDNA synthesis, and quantitative PCR

Cells were lysed by TRIzol reagent (EBL biotechnology, MRE-3200, Taipei, Taiwan) and total RNA was isolated according to the manufacturer’s protocol. cDNA synthesis and quantitative PCR (qPCR) were performed as described in [35]. Primer sequences were listed in Table S1.

### SDS-PAGE and western blot

SDS-PAGE and western blot were performed as previously described [35]. Primary antibodies used in this study were listed in Table S3. Uncropped western blots are presented in Supplemental File.

### Adipokine array

Mouse adipokine array kit (R&D Systems, ARY013) was used to profile mouse adipokines using mouse sera. The assay was performed according to the manufacturer’s instruction.

### Enzyme-linked immunosorbent assay (ELISA)

DuoSet ELISA system was performed to measure adipokines in hAd-CM (R&D Systems, DY1065, DY398, DY1786). The procedure was adapted from the manufacturer’s manual. Briefly, capture antibodies were pre-coated in 96-well uncoated plates. Samples were applied to the coated plates and the detection antibodies, streptavidin-HRP, and NeA-Blue Tetramethylbenzidine substrate (Clinical Science Products, 01016-1-500, Mansfield, MA, USA) were added sequentially. The absorbance was measured at 450/540 nm using SpectraMax Plus 384 (Molecular Devices). For the evaluation of MDA-MB-231 cells after hAd-CM pre-treatment, cells were further cultured in the DMEM for 24 h.

### Immunohistochemistry

Tumor tissue from mice were fixed with 10% formalin and labeled with Serpine1 primary antibody (Table S3). Slides were viewed using 40×/100× objective on a BX51 microscopy system (Olympus, Tokyo, Japan). Serpine1 staining was quantified using the “IHC_Toolbox” plugin of Image J. Tumor sections from 3 mice were analyzed in each group.

### Subcellular fractionation

The procedure of subcellular fractionation was as previously described [35].

### Immunofluorescence staining and imaging

γH2AX foci detection was done according to the previous study [36]. Antibodies were listed in Table S3. Slides were viewed using a 63X objective on an IX71 inverted fluorescence microscopy system (Olympus). Images were collected and processed using ImageJ and cells contained more than 5 foci were counted. Approximately 400–500 cells were counted in each group per test.

For staining of SERPINE1, cells were fixed by 1:1 mixture of methanol and ethanol. Slides were viewed on a Leica TCS SP5 confocal microscope, and 3D images were processed using Imaris software.

### Lentivirus production and transduction

Lentivirus was produced and transduced as previously described [35].

### Cell cycle analysis

Cells were fixed with 70% ice-cold ethanol at indicated time point post-radiation, and the cell cycle distribution was evaluated by propidium iodide (PI) (Sigma, P4170) staining and analyzed by FlowJo software.

### Neutral comet assay

Neutral comet assay was performed with a CometAssay® Kit (TREVIGEN, 4250-050-K, Minneapolis, MN, USA) according to manufacturer’s instruction. Briefly, 1 × 10^5^ cells were resuspended in 1 mL ice-cold PBS (Mg^2+^, Ca^2+^-free) and mixed 10 μL of cell suspension with 50 μL of 1% low-melting point agarose and coated on slides. After solidification, the mixture was lysed and subjected to electrophoresis (35 V, 35 min) on ice. Slides were stained with SafeView^TM^ Classic nucleic acid dye (abm, G108, Richmond, BC, Canada) and then imaged using an IX71 inverted fluorescence microscopy system (Olympus). Tail moment was analyzed by OpenComet software. Approximately 100 comets were counted in each group per test.

### RNA-sequencing and dataset analysis

RNA-sequencing (RNA-seq) was performed on Illumina NovaSeq 6000 using 150 bp paired-end sequencing, and data generated in this study are publicly available in the Gene Expression Omnibus (GEO) database (GSE209894). Sequences were trimmed by Trimmomatic (v0.38) and aligned to the human reference genome (GRCh38) using HISAT2 (v2.1.0). Gene expression was assessed by FeatureCounts (v2.0.0). Genes in the hAd-CM pre-treated groups with a greater than 1.5-fold increase were identified as the differentially expressed genes (DEGs). DEGs were then subject to Database for Annotation, Visualization and Integrated Discovery software (DAVID) and Gene Ontology (GO), KEGG gene sets were applied for pathway analysis. mRNA expression profiles of breast cancer cell lines were obtained from Cancer Cell Line Encyclopedia (CCLE). For clinical data analysis, datasets from the Metastatic Breast Cancer project (METABRIC) [37, 38] were used. In METABRIC project, tumor tissues were obtained from a total of 1904 patients ranging from 26 to 96 years old. GSE76124 [39] and GSE127789 [40] obtained from GEO were also used for *SERPINE1* expression analysis. Tumor tissues were obtained from a total of 151 TNBC patients ranging from 26 to 87 years old (GSE76124).

### Statistical analysis

Two-tailed Student’s *t-test* and one-way ANOVA were performed to analyze the statistical significance. Kaplan–Meier estimate was used to assess the overall survival among clinical datasets. *p < 0.05, **p < 0.01, ***p < 0.001. Error bars of quantitative data were shown as mean ± SD, at least three independent tests were performed for each experiment, unless otherwise mentioned. Graphs were plotted by GraphPad Prism 9.

## Results

### Diet-induced obesity elicits radioresistance in TNBC

**Fig. 1:**
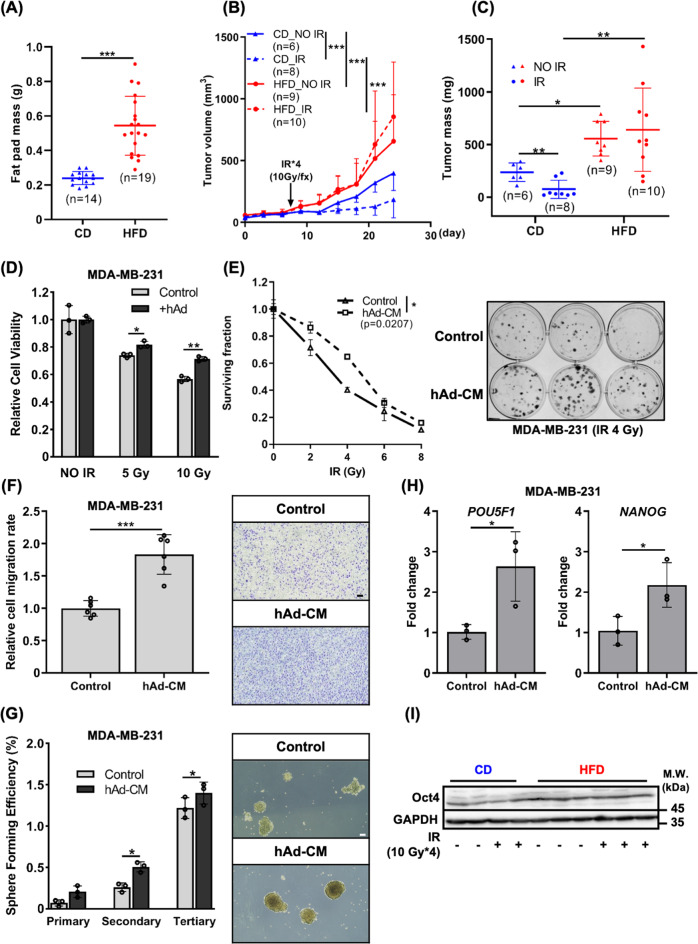
Obesity-mimicking conditions elicited radioresistance and aggressive phenotypes in TNBC. **A** Weight of iWAT excised from the mice fed with CD or HFD. **B**, **C** Tumor volume was measured every 3 days post-tumor implantation and mass was assessed after euthanasia. **D** Cell viability of MDA-MB-231 cells co-cultured with hAd for 3 days followed by IR was assessed by ACP assay. **E** Clonogenicity of MDA-MB-231 cells cultured with control medium or hAd-CM followed by IR. Representative image of colony formation after 4 Gy exposure was shown on the right panel. The migration and stemness capacity of MDA-MB-231 cells pre-treated with control medium or hAd-CM for 3 days were determined by transwell migration assay (**F**) and tumorsphere formation assay (**G**). Representative images were shown on the right panel. Scale bar: 100 μm. **H** mRNA expression of pluripotent genes, *NANOG* and *POU5F1*, were detected by qPCR. **I** Western blotting of Oct4 expression in tumors collected from the mice fed with CD or HFD.

To investigate whether obesity impacts tumor response to RT, we established a DIO syngeneic mouse model of TNBC to receive RT. Female C57BL/6 mice were randomly grouped and fed with either control diet (CD) or high-fat diet (HFD) throughout the experiment. At week 4, EO771 murine TNBC cells were subcutaneously implanted into the right hindlimbs of mice, and fractionated IR for a total of 40 gray (Gy) was administered one-week post tumor injection (Fig. S1A). DIO could already be observed by week 4 and aggravated by week 8. HFD caused significant weight gain while there was no difference in food intake between two groups (Fig. S1B, C). Fasting insulin and glucose levels were significantly elevated in the HFD group than the CD group (Fig. S1D, E). The mass of inguinal white adipose tissue (iWAT) in the mice fed with HFD was also significantly higher than that in the CD group (Fig. 1A). These results indicated that mice exhibited the consequences of DIO including adiposity and hyperinsulinemia, which are two of the major manifestations associated with obesity and metabolic syndrome. Our results showed that DIO significantly accelerated tumor growth. In addition, while IR suppressed tumor growth in the mice fed with CD, tumors in the mice fed with HFD did not respond to IR and tended to grow even faster (Fig. 1B, C). Next, we established an in vitro human white adipocyte differentiation model to further investigate obesity-associated radioresistance (Fig. S1F). After human preadipocytes (hPAd) underwent differentiation and became mature adipocytes (hAd), the maturation of hAd was confirmed by the presence of lipid droplets (Fig. S1G), the increased expression (Fig. S1H) and secretion (Fig. S1I) of two major adipokines, adiponectin and leptin. Recapitulating the observation in vivo, the human TNBC cell line, MDA-MB-231 exhibited reduced sensitivity to IR when co-cultured with hAd (Fig. 1D). These results suggested that obesity not only promoted tumor progression but also took part in modulating the radiosensitivity of TNBC.

### Adipocyte-conditioned medium promotes aggressive phenotypes and radioresistance in TNBC

HFD-induced adiposity often results in increased secretion of adipokines. To test whether the adipocyte-secreted factors impact the radiosensitivity of TNBC, human adipocyte-conditioned medium (hAd-CM) obtained from mature hAd was used (Fig. S1F). Culture with hAd-CM enhanced clonogenic survival and cell viability of multiple TNBC cell lines including MDA-MB-231, HS578T, MDA-MB-468, and BT20 in response to IR (Figs. 1E, S2A, B). Moreover, TNBC cells cultured with hAd-CM exhibited higher migratory ability (Figs. 1F, S2C), stemness capacity (Fig. 1G), and an increase in the expression of pluripotent transcription factors, *NANOG* and *POU5F1* (Oct4), which was also observed in the tumors of DIO mice (Fig. 1H–I). Altogether, these data indicated that factors secreted by adipocytes promote aggressive phenotypes and radioresistance of TNBC cells.

### SERPINE1 is elevated in the circulation and is enriched in the tumor microenvironment of DIO mice

**Fig. 2:**
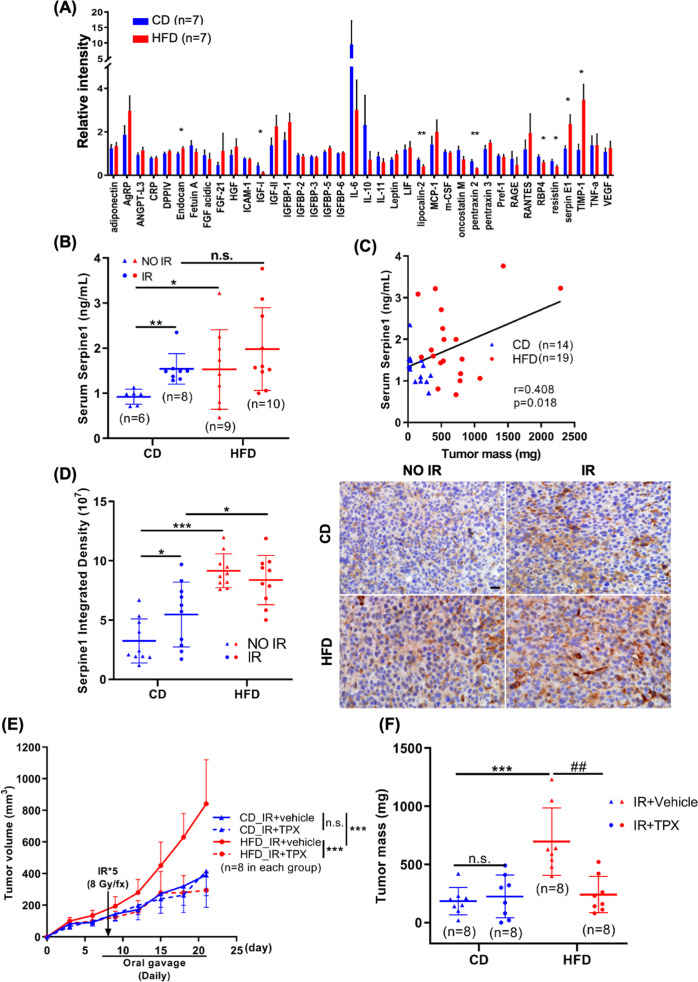
SERPINE1 was increased in both circulation and tumors of DIO syngeneic TNBC mice. **A** Sera from C57BL/6 female mice fed with CD or HFD for 4 weeks were pooled and subjected to an adipokine array to identify the candidate adipokines that were related to DIO. *N* = 7 in each group. **B** Serum level of Serpine1 from both CD and HFD groups of mice were measured by ELISA. **C** Correlation between Serpine1 level and tumor weight in the mice fed with CD or HFD. **D** IHC staining of Serpine1 level in the tumor sections in each group of mice. 10 fields from 3 tumor sections were analyzed for each group. Representative images were shown on the right panel. Scale bar: 20 μm. **E**, **F** Tumor volume was measured every 3 days post-tumor implantation, and mass was assessed after euthanasia. TPX: tiplaxtinin.

An adipokine array was performed to find out the potential circulating adipokines that contributed to the radioresistance of TNBC in DIO mice. Among the 38 adipokines screened in the array, the levels of Endocan, Serpine1, and Timp1 were significantly increased in the sera of DIO mice (Fig. 2A, B). SERPINE1 is tightly correlated with obesity, and the increased serum level of SERPINE1 is associated with higher incidence of metabolic syndrome [23, 41]. It has been well known that adipocytes secrete SERPINE1 [42]. Indeed, the expression and secretion levels of SERPINE1 in hAd were increased upon differentiation (Fig. S2D, E), and the secretion and protein expression of SERPINE1 were slightly enhanced in MDA-MB-231 cells cultured with hAd-CM (Fig. S2F, G). In our DIO syngeneic mouse model, the serum level of Serpine1 was positively correlated with tumor mass (Fig. 2C), and Serpine1 was also abundant within the tumor microenvironment in HFD groups (Fig. 2D), implying that the adipocyte-secretome including SERPINE1 may enhance SERPINE1 expression and secretion in cancer cells. Tiplaxtinin, a selective and orally efficacious inhibitor of SERPINE1, was applied to test whether SERPINE1 plays a role in DIO-mediated tumor radioresistance (Fig. S2H). The administration of tiplaxtinin significantly resensitized tumors to IR in DIO mice while the tumor growth in mice fed with CD was not affected by SERPINE1 inhibition (Fig. 2E, F). Moreover, body weight of mice was not altered by SERPINE1 inhibition either in CD or in HFD group (Fig. S2I). These results indicate that SERPINE1 plays a main role in promoting tumor radioresistance in DIO mice.

### IR induces SERPINE1 expression and nuclear localization in TNBC cells

**Fig. 3:**
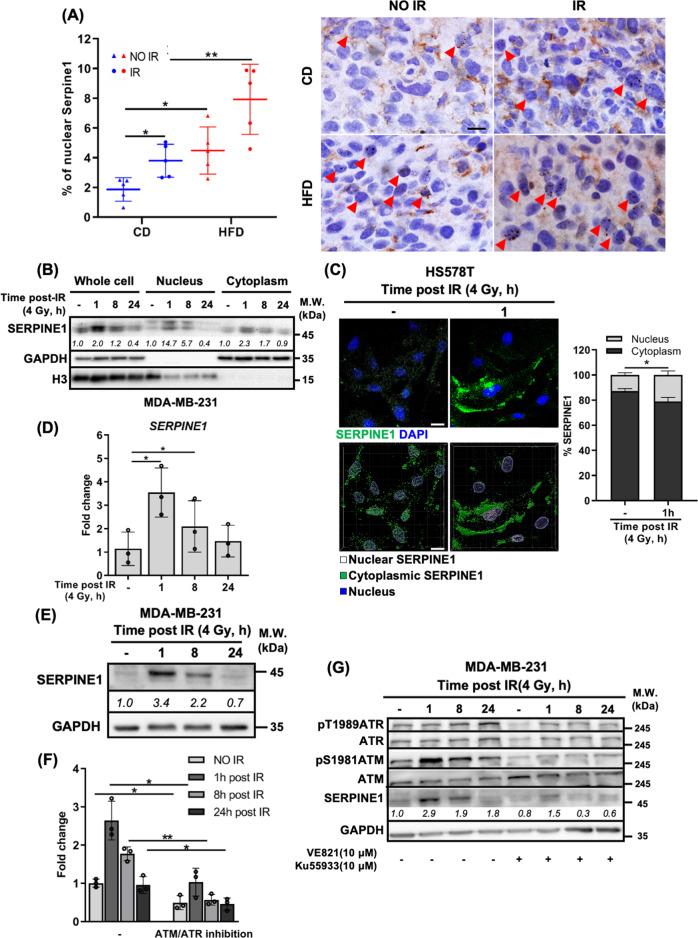
IR induced the expression and nuclear localization of SERPINE1 in TNBC cells. **A** The nuclear Serpine1 in the tumor sections from each group of mice was detected by IHC staining. 5 fields from 3 tumor sections were analyzed for each group. Representative images were shown on the right panel. Scale bar: 10 μm. **B** The subcellular localization of IR-induced SERPINE1 in MDA-MB-231 cells was detected by western blotting following subcellular fractionation. **C** Distribution of intracellular SERPINE1 in HS578T cells post-IR was detected by immunofluorescence staining and imaged by confocal microscopy. Approximately 20 cells were analyzed in each group per test. Representative images were shown on the left panel. 2D images were shown on the top. 3D images were shown on the bottom. Scale bar: 20 μm. mRNA (**D**) and protein (**E**) level of SERPINE1 in MDA-MB-231 cells post-IR were evaluated by qPCR and western blotting, respectively. The kinetic of SERPINE1 induction post-IR in MDA-MB-231 cells treated with 10 μM of ATM and ATR inhibitors were detected by (**F**) qPCR and (**G**) western blotting.

In addition to DIO, we also observed that both circulating and intratumoral Serpine1 were elevated in the mice receiving IR (Fig. 2B, D). Moreover, we found that Serpine1 was increased not only in the cytoplasm but also in the nucleus following IR or HFD, and tumors from DIO mice receiving IR exhibited the highest percentage of nuclear Serpine1 (Fig. 3A). IR-induced nuclear SERPINE1 was also observed in multiple TNBC cell lines and radioresistant BC cells, evidenced by both subcellular fractionation (Figs. 3B, S3A) and immunofluorescence staining with confocal imaging (Figs. 3C, S3B). Furthermore, we found that SERPINE1 was transcriptionally induced in TNBC cells upon IR (Figs. 3D, E, S3C) in an ATM/ATR-dependent manner (Fig. 3F, G). Treating MDA-MB-231 cells with a chemotherapeutic agent, doxorubicin, also induced SERPINE1 expression in an ATM/ATR-dependent manner (Fig. S3D), suggesting that SERPINE1 was induced following the generation of DSBs.

### SERPINE1 plays both cancer cell-autonomous and non-autonomous roles in promoting the aggressiveness and radioresistance of TNBC cells

**Fig. 4:**
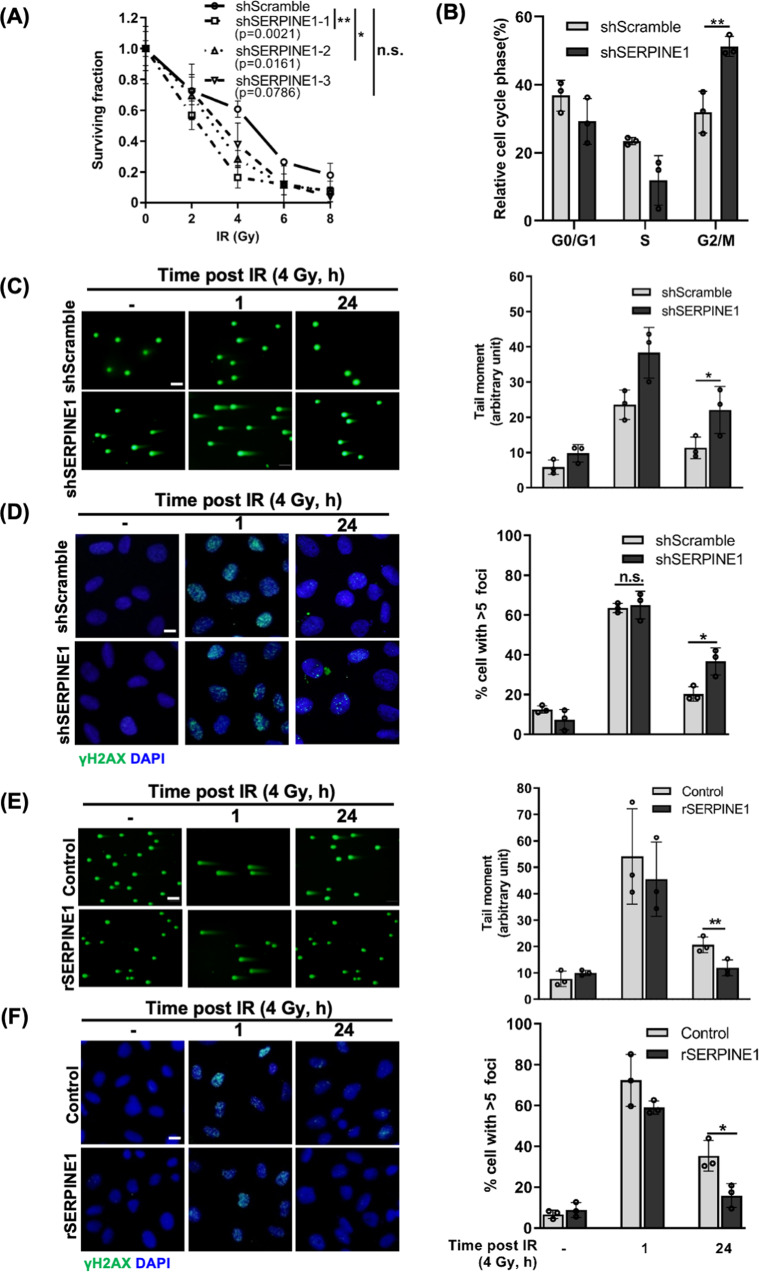
Both intracellular and extracellular SERPINE1 facilitated aggressiveness and DSB repair in TNBC cells. **A** Clonogenicity of *SERPINE1*-knockdown (shSERPINE1-1, -2, -3) and control (shScramble) MDA-MB-231 cell lines followed by IR were evaluated by clonogenic survival assay. **B** Cell cycle distributions between shScramble or shSERPINE1 MDA-MB-231 cells were measured by flow cytometry. **C** DSBs induced by 4 Gy IR in shScramble and shSERPINE1 MDA-MB-231 cells were assessed by neutral comet assay. Approximately 100 comets were counted in each group per test. Representative fluorescence microscope images were shown on the left panel. **D** IR-induced γH2AX foci formation in shScramble and shSERPINE1 cells post 4 Gy IR were measured by immunofluorescence staining. Cells contained more than 5 foci were counted. Approximately 400–500 cells were counted in each group per test. Representative images of γH2AX foci were shown on the left. Scale bar: 20 μm. **E** DSBs induced by 4 Gy IR in control and 10 ng/mL rSERPINE1-treated MDA-MB-231 cells were assessed by neutral comet assay. rSERPINE1 was pre-treated 1 h prior to 4 Gy IR. **F** IR-induced γH2AX foci formation was calculated in the control and the 10 ng/mL rSERPINE1-supplemented MDA-MB-231 cells. rSERPINE1 was pre-treated 1 h prior to 4 Gy IR.

Next, we attempted to explore how SERPINE1 participates in regulating TNBC aggressiveness and radioresistance. Gene expression analysis showed that *SERPINE1* was differentially expressed among different breast cancer subtypes, as defined by molecular features [43]. *SERPINE1* was highly expressed in the basal B subtype, which is a more aggressive subtype of TNBC (Fig. S4A, B). Knockdown of *SERPINE1* rendered MDA-MB-231 cells more susceptible to IR (Figs. 4A, S4C) and abolished tumorsphere formation (Fig. S4D), cancer cell migration (Fig. S4E) and invasion (Fig. S4F), suggesting that SERPINE1 was crucial for maintaining the aggressive phenotypes of TNBC cells. An unresolved G2/M arrest in *SERPINE1*-knockdown cells 24 h post-IR indicated that these cells were less capable to recover from IR-induced cell cycle arrest (Fig. 4B). Indeed, DSBs and γH2AX foci were accumulated in the *SERPINE1*-knockdown MDA-MB-231 cells 24 h post-IR, while most of DSBs had been repaired in the control cells (Fig. 4C, D). Furthermore, treating MDA-MB-231 cells with recombinant SERPINE1 (rSERPINE1) elicited more efficient DSB repair (Fig. 4E, F), demonstrating that extracellular SERPINE1 also plays a role in regulating IR-induced DSB repair. Together with the results shown in Fig. 3, we have identified a non-canonical role of SERPINE1 in facilitating DSB repair, which further contributes to the resistance of cancer cells to IR.

**Fig. 5:**
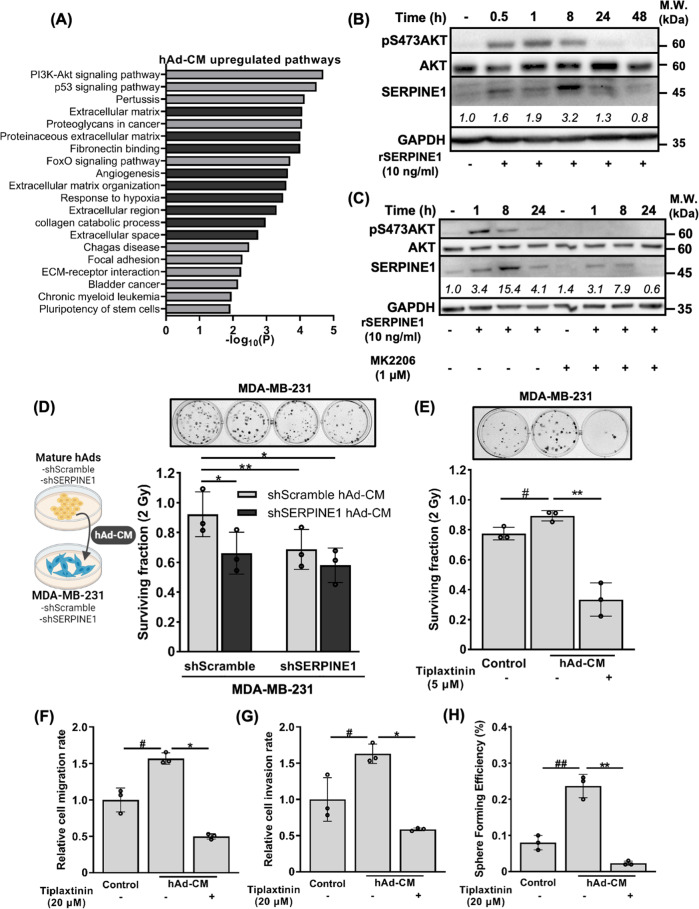
SERPINE1 activated AKT signaling and contributed to radioresistance and aggressive phenotypes of MDA-MB-231 cells cultured with hAd-CM. **A** Pathway analysis using DAVID bioinformatics database. Top 20 upregulated biological pathways categorized by KEGG (light gray) or GO (dark gray) in MDA-MB-231 cells cultured with hAd-CM. **B**, **C** AKT phosphorylation and SERPINE1 induction in MDA-MB-231 cells treated with 10 ng/mL rSERPINE1 and 1 μM MK2206 were detected by western blotting. MK2206 was pre-treated 1 h prior to rSERPINE1 supplement. Clonogenicity of *SERPINE1*-knockdown MDA-MB-231 cells cultured with indicated hAd-CM (**D**) or parental MDA-MB-231 cells cultured with hAd-CM containing DMSO or 5 μM Tiplaxtinin (**E**) for 3 days followed by IR. Surviving fraction upon 2 Gy IR exposure was quantified. Representative images were shown above. The migratory capacity (**F**), invasiveness (**G**), and stemness status (**H**) of MDA-MB-231 cells cultured with hAd-CM containing DMSO or 20 μM Tiplaxtinin for 3 days were assessed by transwell assay and tumorsphere formation assay.

Our RNA-sequencing analysis showed that PI3K/AKT signaling pathway was significantly upregulated in the MDA-MB-231 cells cultured with hAd-CM (Fig. 5A), and SERPINE1 has been reported to induce AKT signaling pathway [28, 44]. We found that AKT phosphorylation and intracellular SERPINE1 expression were concomitantly induced by the administration of rSERPINE1 to MDA-MB-231 cells (Fig. 5B). Moreover, treating cells with an AKT inhibitor, MK2206, showed that cancer cell-autonomous SERPINE1 expression was upregulated in an AKT-dependent manner (Fig. 5C).

According to our DIO model, the mice fed with HFD exhibited hyperinsulinemia (Fig. S1D). Insulin has been reported to correlate with Serpine1 expression in vivo [23, 45]. Upon the treatment of insulin to MDA-MB-231 cells, AKT phosphorylation and SERPINE1 expression were induced concomitantly (Fig. S5A). Moreover, the combination of SERPINE1 and insulin enabled TNBC cells to become more radioresistant (Fig. S5B). The expression of PLAUR, one of the known receptors of SERPINE1 [46], was increased in ER-negative breast tumors and was slightly enhanced in the basal B subset (Fig. S5C–E). However, knockdown of *PLAUR* in MDA-MB-231 cells did not affect SERPINE1-mediated AKT activation and the induction of intracellular SERPINE1 (Fig. S5F, G). Therefore, we excluded PLAUR to be a potential receptor involved in extracellular SERPINE1-mediated signaling.

### SERPINE1 is critical for establishing radioresistance of TNBC cells under obesity-mimicking condition

Following our findings on both intracellular and extracellular SERPINE1 may be important for DSB repair in cancer cells, we attempted to deplete SERPINE1 in MDA-MB-231 cells or in hAd to obtain hAd-CM with low level of SERPINE1 (Fig. S6A) to clarify whether cancer cell autonomous or non-autonomous SERPINE1 is important for establishing radioresistance. Depleting SERPINE1 in both hAd-CM and MDA-MB-231 cells displayed the least surviving fraction of MDA-MB-231 cells following IR, while depleting SERPINE1 in either hAd-CM or MDA-MB-231 cells alone, it only slightly affected the radiosensitivity of MDA-MB-231 cells (Fig. 5D). More evident observations were made when applying tiplaxtinin to the co-culture of hAd-CM and MDA-MB-231 cells (Figs. 5E, S6B). The administration of tiplaxtinin in the co-culture system not only dampened hAd-CM-promoted radioresistance (Fig. 5E) but also largely alleviated multiple hAd-CM-associated aggressive phenotypes such as migration (Figs. 5F, S6C), invasion (Figs. 5G, S6D), and stemness capacity (Figs. 5H, S6E). In conclusion, SERPINE1 secreted by hAd and expressed in MDA-MB-231 cells contribute to the development of radioresistance under obesity-mimicking condition.

### SERPINE1 level was higher in TNBC patients and predicts patient outcome

**Fig. 6:**
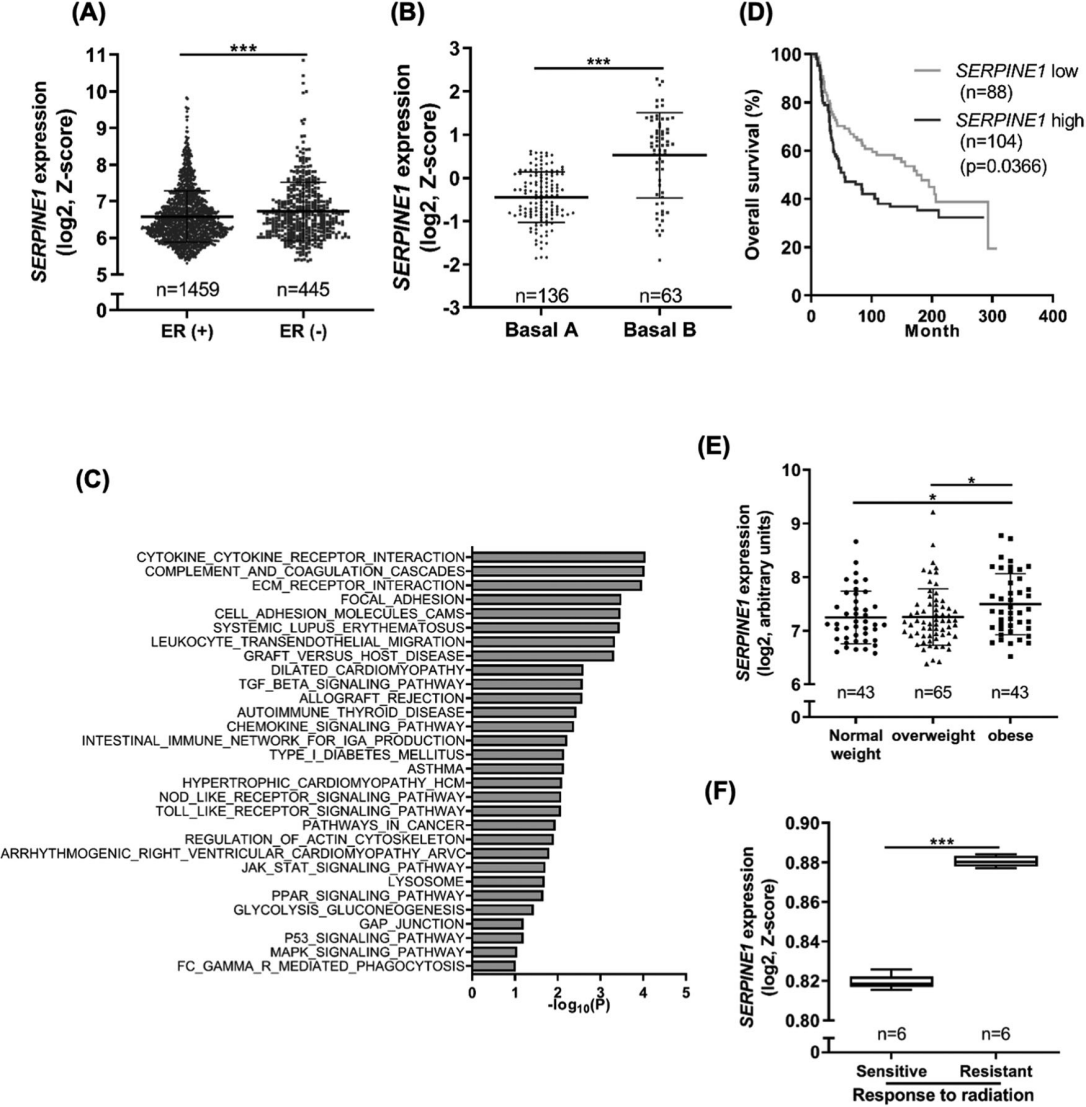
SERPINE1 was positively correlated with worse clinical outcomes. **A**–**D** Analyses of RNA-sequencing data from METABRIC dataset. *SERPINE1* expression in the breast tumors with different ER status (**A**) or TNBC subtypes (**B**). **C** Gene set enrichment analysis (GSEA) using KEGG database. Top 30 upregulated biological pathways among SERPINE1-high group were shown. **D** Overall survival rate of TNBC patients. **E** *SERPINE1* expression level in breast tumor tissues of TNBC patients with different BMI status. Normal weight: BMI < 25; overweight: 25 <BMI < 30; obese: BMI > 30 (GSE76124). **F** *SERPINE1* expression in parental or radioresistant MDA-MB-231 cell lines (GSE127789).

Analysis of gene expression data from public clinical datasets revealed that *SERPINE1* expression was significantly elevated in the ER-negative breast tumors (Fig. 6A), particularly in the basal B subtype (Fig. 6B). In addition, higher *SERPINE1* level was positively correlated with several pathways associated with oncogenic signaling, cell migration and invasion (Fig. 6C). In TNBC, *SERPINE1* expression was increased in the tumors of obese patients (Fig. 6E) and was correlated with worse overall survival (Fig. 6D). Moreover, *SERPINE1* expression (Fig. 6F) was increased in the radioresistant TNBC cell lines compared with the parental cell lines. Altogether, high *SERPINE1* expression may predict tumor aggressiveness and poor patient outcome.

## Discussion

Our results demonstrated that under obese conditions, TNBC is less responsive to RT, and one of the adipocyte-secreted factors, SERPINE1, was identified as a key determinant for the therapeutic response. Mechanistically, circulating SERPINE1 or IR induces the expression of intracellular SERPINE1 in TNBC cells. Cancer cell-autonomous SERPINE1 can be either secreted to the tumor microenvironment to reinforce SERPINE1-mediated signaling or translocated to nucleus to facilitate DSB repair. The combinatorial effects of SERPINE1 reprogram TNBC cells to become more aggressive and radioresistant.

Systemic metabolic disorders, including high BMI level and diabetes are risk factors for cancers, particularly breast cancer [47]. Excess adipose tissue influences tumor progression through hormonal and non-hormonal mechanisms, while the latter is more predominant in regulating TNBC due to the absence of hormone receptors. Both central and local fats contribute to a tumor-favorable environment via autocrine or paracrine effects mediated by adipocyte-secreted factors [8, 48]. Several adipokines have been shown to trigger oncogenic signaling pathways. For example, the elevated levels of leptin and resistin under obese conditions are reported to facilitate cell proliferation, metastasis, stemness, or alter intratumoral oxidative status and the components of immune system both in vitro and in vivo [8, 49, 50]. In addition, hyperinsulinemia or chronic insulin stimulation occurs in obese individuals is associated with tumor progression in TNBC by altering mitochondrial activity, histone modification, and several oncogenic signaling pathways [48, 51, 52]. Moreover, these adipocyte-secreted factors can also induce SERPINE1 level to cooperatively promote cancer progression [45, 53]. These reports support that the combinatorial crosstalk among adipocytes, cancer cells, and tumor microenvironment regulates tumor progression and therapeutic response.

We demonstrated that the alteration of systemic metabolism in DIO perturbs tumor microenvironment to promote both tumor growth and radioresistance. Furthermore, based on the results from the adipokine array, we targeted SERPINE1 as a central hub linking obesity, cancer progression, and radioresistance in TNBC. The balance of SERPINE1 level is critical for maintaining physiological processes and SERPINE1 is often deregulated in various pathological states. For example, SERPINE1 plays a role in the plasminogen activator system to regulate hemostasis [22, 46]. It is also regarded as an adipogenic factor tightly associated with metabolic syndrome [23, 41]. In addition, SERPINE1 plays a predominant role in cancer progression, including regulations of EMT, stemness, metabolic reprogramming, and therapeutic resistance [27–29, 44, 54]. Both adipocytes and cancer cells can promote tumor metastasis after receiving the exogenous or inducing the cell-autonomous SERPINE1 in the co-culture system, indicating that adiposity can facilitate the SERPINE1-mediated metastasis [53, 55]. Indeed, based on our results from the transwell migration assay, the obesity-mimicking condition was able to promote migration of TNBC cells, and the phenomenon was dampened after SERPINE1 depletion, pinpointing a critical role for SERPINE1 in obesity-promoted cancer cell migration (Fig. 5). In addition to the well-established characteristics of SERPINE1 on cancer progression, in our study, we demonstrated that SERPINE1 is a crucial factor linking obesity to tumor radioresistance in several potential mechanisms. The dysregulated lipid metabolism has been shown to link with a higher level of acetyl-CoA pool and promotes histone acetylation both in vitro and in vivo [51, 56]. Acetylation of certain genes further leads to promoting cancer stemness, metastatic capacity, and therapeutic resistance [57]. Our RNA-sequencing data revealed that AKT signaling was highly enhanced under obesity-mimicking conditions in TNBC cells. Also, when treated cells with SERPINE1 and insulin, the activation of AKT further induced SERPINE1 expression in a positive-feedback manner, indicating that SERPINE1 might modulate cancer aggressiveness under the control of AKT signaling in obesity (Fig. 5). Moreover, we also identified a novel role of SERPINE1 in DSB repair (Figs. 3 and 4). A paralog of SERPINE1, SERPINE2, has been recently reported to participate in DNA repair process through the direct interaction with ATM and MRE11 to protect cancer cells from IR-induced DNA damage [58]. Moreover, due to the highly conserved sequence between the carboxyl-terminus of SERPINE1 and the ATM/MRE11 binding regions on SERPINE2, it is plausible that SERPINE1 directly interacts with ATM/MRN axis to initiate DDR and to facilitate IR-induced DSB repair. Also, we found that SERPINE1 was induced and translocated into nucleus upon IR, supporting that SERPINE1 is directly involved in DDR and contributes to the development of radioresistance in TNBC cells.

Due to the complexity of the tumor microenvironment in obesity, it is likely that other factors may also participate in the regulatory network of obesity-promoted tumor radioresistance. Another adipogenic marker, TIMP1 (tissue inhibitor of matrix metalloproteinase 1), was also increased in the circulation of DIO mice. TIMP1 has been reported to promote cell proliferation in TNBC and can modulate the radiosensitivity of cancer cells based on its enzymatic inhibition characteristics [59–61]. Whether TIMP1 plays a role in coordination with SERPINE1 in this scenario warrants further investigation.

Our hAd-CM model, albeit simple and limited to only hAd-secreted factors, revealed that hAd-CM-promoted radioresistance and aggressiveness of TNBC were dampened when SERPINE1 in both hAd and cancer cells were depleted genetically or pharmacologically (Fig. 5), suggesting that both the adipocyte-secreted and the cancer cell-autonomous SERPINE1 were important to contribute to the aggressive phenotypes. In our in vivo model, the radioresistance in DIO mice was significantly ameliorated when a selective SERPINE1 inhibitor was applied, further demonstrating an indispensable role of SERPINE1 in the obese tumor microenvironment in promoting tumor radioresistance. In addition, the increased SERPINE1 secretion from cancer cells has been reported to influence the adjacent cancer cells or immune cells, further causing tumors to become more resistant to radio- and chemo-therapies [28, 30]. Moreover, SERPINE1 can be produced and secreted by macrophages and fibroblasts [62, 63], which are also critical components in the tumor microenvironment, warranting further investigation.

In addition to the cancer cell-autonomous SERPINE1, the circulating SERPINE1 is also important and requires receptors to exert signaling in cancer cells. Although PLAUR is the most well-characterized receptor for SERPINE1 in both physiological and pathological states [46], our result showed that SERPINE1 activated the downstream pathways independent of PLAUR (Fig. S5). Other receptors may be involved in SERPINE1-mediated responses. For example, very low density lipoprotein receptor (VLDLR) and the low-density lipoprotein receptor related protein 1 (LRP-1) are reported as receptors of SERPINE1, and are also associated with radioresistance in TNBC [54, 64].

In conclusion, we demonstrate the impacts of obese conditions on TNBC progression and responsiveness to IR both in vitro and in vivo. Moreover, we reveal that SERPINE1 plays an indispensable role in obesity-associated radioresistance. Based on our analysis of the clinical datasets, SERPINE1 is enriched in TNBC patients, particularly those who are obese, and is associated with poor prognosis, suggesting that SERPINE1 can be a promising prognostic marker. Furthermore, SERPINE1 may also be developed as a therapeutic target to overcome obesity-associated radioresistance in TNBC.

## Supplementary information

Extended Data

Reproducibility checklist

Original Uncropped Western blots

## Data Availability

Data generated in this study are publicly available in Gene Expression Omnibus (GEO) database (GSE209894).
