# Supplementary material for: Obesity promotes radioresistance through SERPINE1-mediated aggressiveness and DNA repair of triple-negative breast cancer
Source: Cell Death Dis. 2023 Jan 21;14(1):53. doi: 10.1038/s41419-023-05576-8 (PMC9867751; doi:10.1038/s41419-023-05576-8)
Supplement: Supplementary file 1 — Extended Data [file 41419_2023_5576_MOESM1_ESM.zip › Supplementary data_Su&Kuo_R1/Supplementary Data_clean_Su-Kuo_Proofread_ESM.docx]

**Obesity promotes radioresistance through SERPINE1-mediated aggressiveness and DNA repair of triple-negative breast cancer**

**Yong-Han Su, Yi-Zhen Wu, David K. Ann, Jenny Ling-Yu Chen and Ching-Ying Kuo**

**Supplementary Information**

**SUPPLEMENTARY TABLES**

**Supplementary Table S1. List of qPCR primers**

| **Gene** | **Forward (5’-3’)** | **Reverse (5’-3’)** |
| --- | --- | --- |
| *NANOG* | CTCCAACATCCTGAACCTCAGC | CGTCACACCATTGCTATTCTTCG |
| *POU5F1* | CCTGAAGCAGAAGAGGATCACC | AAAGCGGCAGATGGTCGTTTGG |
| *SERPINE1* | TGCCCATGATGGCTCAGA | GCAGTTCCAGGATGTCGTAGTAATG |
| *18S* | CGGCGACGACCCATTCGAAC | GAATCGAACCCTGATTCCCCGTC |

**Supplementary Table S2. List of PCR primers**

| **Gene** | **Forward (5’-3’)** | **Reverse (5’-3’)** |
| --- | --- | --- |
| *ADIPOQ* | ATGCTGTTGCTGGGAGCTGTTC | CCACACTGAATGCTGAGCGGTA |
| *LEP* | GAACCCTGTGCGGATTCTTG | CACCTCTGTGGAGTAGCCTGAA |
| *SERPINE1* | GGGTGTTTCAGCAGGTGGCG | CTGGAGTCGGGGAAGGGAGT |
| *18S* | same as qPCR primers | |

**Supplementary Table S3. List of antibodies**

| **Target protein** | **Catalog number** | **Manufacturer** | **Location** |
| --- | --- | --- | --- |
| SERPINE1  (for Western blotting/ IHC) | 612024 | BD Biosciences | Franklin Lakes, NJ, USA |
| SERPINE1 (for IF staining) | sc-5297 | Santa Cruz | Dallas, Texas, USA |
| pS139H2AX | 2577 | Cell signaling | Danvers, MA, USA |
| pS473AKT | 9271 | Cell signaling |  |
| AKT | 9272 | Cell signaling |  |
| PLAUR | A1397 | ABclonal | New Taipei City, Taiwan |
| pT1989ATR | 30632 | Cell signaling |  |
| ATR | 13934 | Cell signaling |  |
| pS1981ATM | ab81292 | abcam | Cambridge, UK |
| ATM | ab78 | abcam |  |
| Oct4 | 11263-1-AP | Proteintech | Rosemont, IL, USA |
| GAPDH | 60004-1-lg | Proteintech |  |
| Tubulin | 66031-1-lg | Proteintech |  |
| H3 | 17168-1-AP | Proteintech |  |

**SUPPLEMENTARY MATERIALS AND METHODS**

**Neutral lipid droplet staining**

Neutral lipid droplets were stained by BODIPY 493/503 (Cayman, 25892), according to a protocol modified from the previous publication (1). Briefly, cells seeded in a 12-well plates were washed once with PBS and stained with 1 μM of PBS-diluted BODIPY at 37 °C for 15 minutes.

**Polymerase chain reaction**

PCR was performed using DreamTaq Green PCR master mix (Thermo Fisher Scientific, K1081, Waltham, MA, USA) on Primus 25 thermocycler (PeqLab, Darmstadt, Germany). Primer sequences were listed in Table S2.

**SUPPLEMENTARY FIGURES**

**Figure S1. Mice fed with HFD manifested diet-induced obesity and hyperinsulinemia.**

**A** Schematic diagram for the experimental design of the DIO syngeneic mouse model of TNBC. **B, C** Food intake and body weight of mice fed with control diet (CD) or high fat diet (HFD) were measured routinely. **D, E** Measurement of the serum fasting insulin **(D)** and fasting glucose level **(E)** from both groups of mice at the indicated time points. **F** A schematic diagram of human preadipocytes (hPAd) differentiation and collection of hAd-CM. **G** BODIPY 493/503 staining of neutral lipid droplets in mature adipocytes on day 15 post-differentiation. Scale bar: 20 µm. **H, I** Expression and secretion profiles of adipogenic factors during hAd differentiation were assessed by RT-PCR **(H)** and ELISA **(I)**, respectively.

**Figure S2. Adipocyte-secreted factors promoted the aggressiveness and the radioresistance of TNBC cells.**

**A** Clonogenicity of HS578T cells cultured with control medium or hAd-CM followed by IR was evaluated by clonogenic survival assay. Representative image of colony formation after 2 Gy exposure was shown below. **B** Cell viability of hAd-CM-cultured MDA-MB-468 and BT20 cells after IR exposure were assessed by ACP assay. **C** The migration of MDA-MB-468 and BT20 cells cultured with control medium or hAd-CM for 3 days were measured by transwell migration assay. **D, E** Expression and secretion profiles of SERPINE1 during hAd differentiation were assessed by RT-PCR **(D)** and ELISA **(E)**, respectively. **F, G** Secretion level and protein level of SERPINE1 in MDA-MB-231 cells cultured with control medium or hAd-CM was measured by ELISA **(F)** and western blotting **(G)**, respectively. **H** Schematic diagram for the experimental design of the tiplaxtinin-treated DIO syngeneic mouse model. **I** Body weight of mice was measured routinely. TPX: tiplaxtinin.

**Figure S3. Intracellular SERPINE1 in TNBC cells was induced and redistributed subcellularly under genotoxic stress.**

**A** The cellular localization of IR-induced SERPINE1 in MDA-MB-436 cells was detected by subcellular fractionation. **B** Distribution of intracellular SERPINE1 in radioresistant MDA-MB-231 cells was detected by immunofluorescence staining and imaged by confocal microscopy. 2D images were shown on the top and 3D mode of images were shown on the bottom. Scale bar: 20 µm. **C** SERPINE1 induction post-IR in MDA-MB-436 and BT20 cells were determined by western blotting. **D** SERPINE1 induced by doxorubicin in MDA-MB-231 cells was assessed by western blotting. Phosphorylation of ATM or ATR was inhibited by Ku55933 or VE821 2 hours prior doxorubicin treatment, respectively.

**Figure S4. SERPINE1 was highly expressed in TNBC cells and was critical for maintaining the aggressiveness of cancer cells.**

**A** mRNA expression level of *SERPINE1* was obtained from the Cancer Cell Cline Encyclopedia (CCLE). Cell lines were categorized by molecular subtyping. **B** Protein expression levels of SERPINE1 in different subtypes of BC cell lines were determined by western blotting. **C** Knockdown efficiency of *SERPINE1* in MDA-MB-231 cells was assessed by western blotting. **D** The stemness status of shScramble and shSERPINE1 cells were assessed by tumorsphere formation assay. **E, F** Migratory capacity **(E)** and invasiveness **(F)** of shScramble and shSERPINE1 cells were evaluated by transwell assay. Representative images were shown above. Scale bar: 100 µm.

**Figure S5. The exogenous SERPINE1-mediated regulation on MDA-MB-231 cells was PLAUR-independent.**

**A** Time course of SERPINE1 induction in MDA-MB-231 cells treated with recombinant insulin was determined by western blotting. **B** Clonogenicity of MDA-MB-231 cells pre-treated with recombinant SERPINE1 and insulin for 1 hour followed by IR were evaluated by clonogenic survival assay. Representative image of colony formation after 2 Gy exposure was shown below. **C** *PLAUR* expression level in BC patients was obtained from METABRIC dataset. Patients were grouped according to their ER status. **D** mRNA expression level of *PLAUR* was obtained from Cancer Cell Cline Encyclopedia (CCLE). Cell lines were categorized by molecular subtyping. **E** Protein expression levels of PLAUR in different subtypes of BC cell lines were determined by western blotting. **F** Knockdown efficiency of PLAUR in MDA-MB-231 cells was assessed by western blotting. **G** AKT phosphorylation and SERPINE1 induction in shScramble or shPLAUR MDA-MB-231 cells treated with recombinant SERPINE1 were assessed by western blotting.

**Figure S6. SERPINE1 contributed to radioresistance and aggressive phenotypes of MDA-MB-231 cells cultured with hAd-CM.**

**A** Reduction of the SERPINE1 secretion from *SERPINE1*-knockdown hAds was confirmed by ELISA. **B** Susceptibility of tiplaxtinin in MDA-MB-231 cells was evaluated by ACP assay. **C-E** Representative images of the migratory capacity **(C)**, invasiveness **(D)**, and stemness status **(E)** of MDA-MB-231 cells pre-treated with hAd-CM containing DMSO or 20 μM Tiplaxtinin for 3 days. Scale bar: 100 μm.

**REFERENCES**

1. Qiu B, Simon M. BODIPY 493/503 Staining of Neutral Lipid Droplets for Microscopy and Quantification by Flow Cytometry. BIO-PROTOCOL. 2016;6(17).
