# Supplementary figures and images for: Obesity promotes radioresistance through SERPINE1-mediated aggressiveness and DNA repair of triple-negative breast cancer
Source: Cell Death Dis. 2023 Jan 21;14(1):53. doi: 10.1038/s41419-023-05576-8 (PMC9867751; doi:10.1038/s41419-023-05576-8)

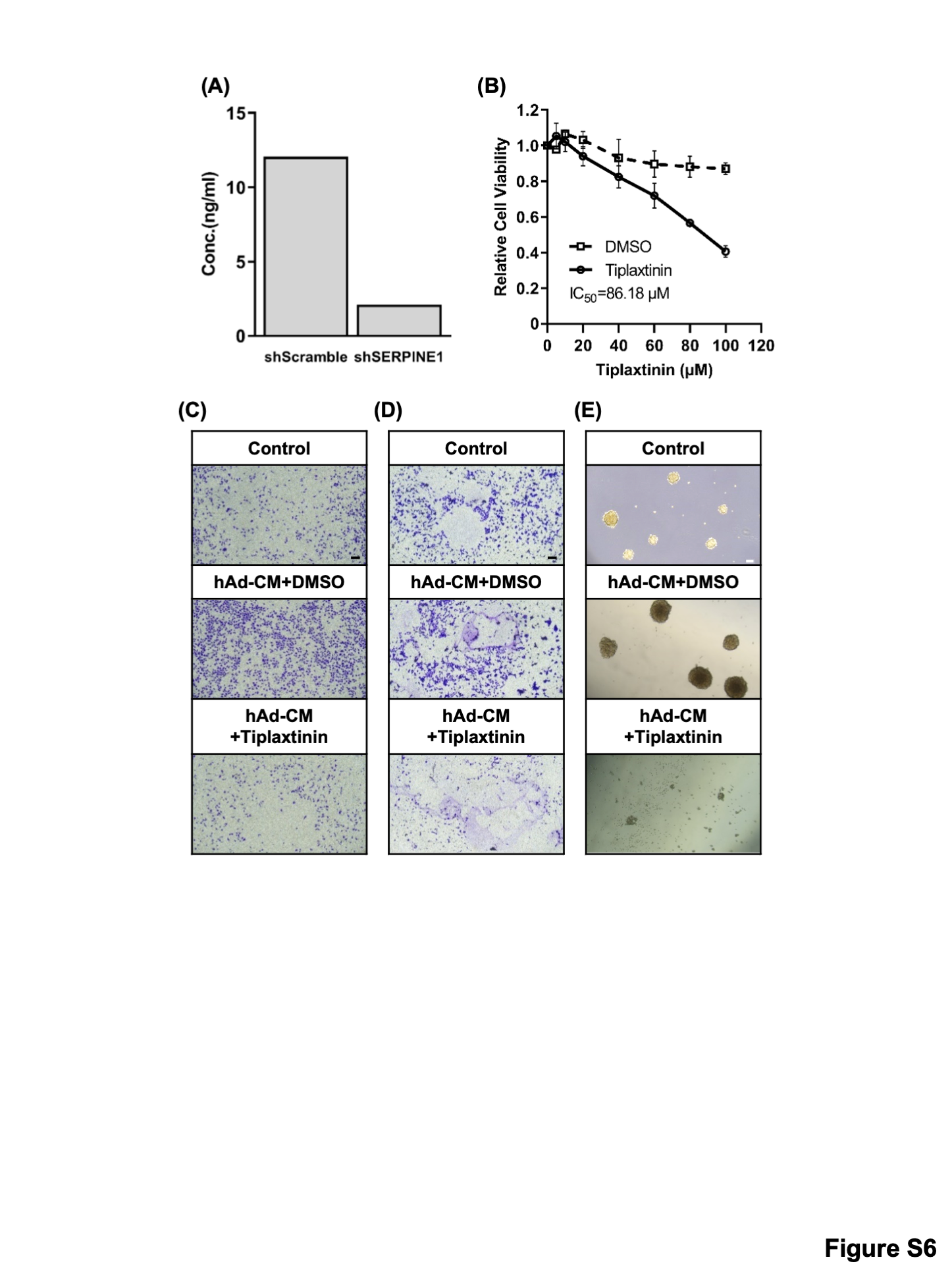

Supplement: Supplementary file 1 — Extended Data [file 41419_2023_5576_MOESM1_ESM.zip › Supplementary data_Su&Kuo_R1/Fig. S6.tiff]

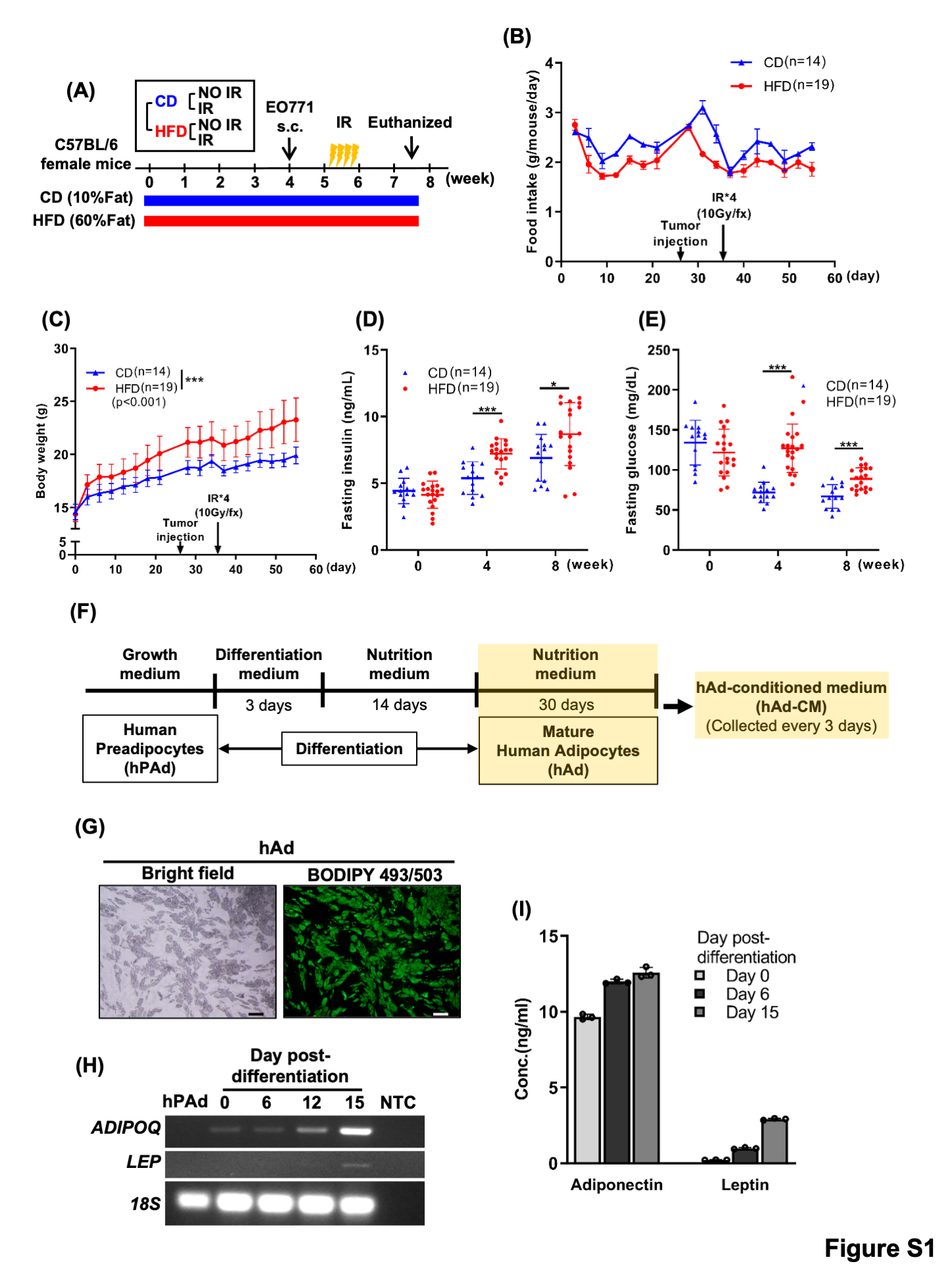

Supplement: Supplementary file 1 — Extended Data [file 41419_2023_5576_MOESM1_ESM.zip › Supplementary data_Su&Kuo_R1/Fig. S1.tiff]

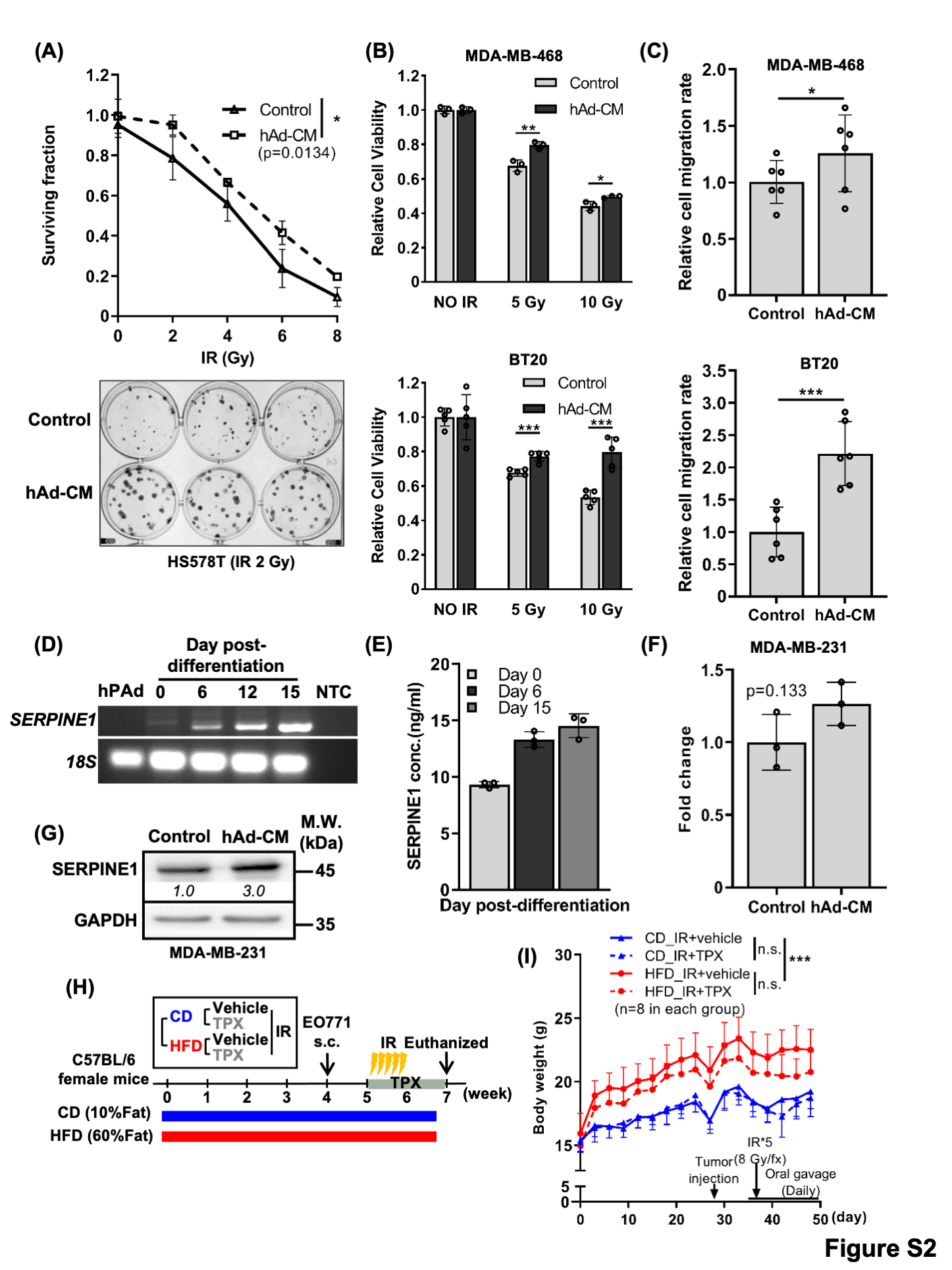

Supplement: Supplementary file 1 — Extended Data [file 41419_2023_5576_MOESM1_ESM.zip › Supplementary data_Su&Kuo_R1/Fig. S2.tiff]

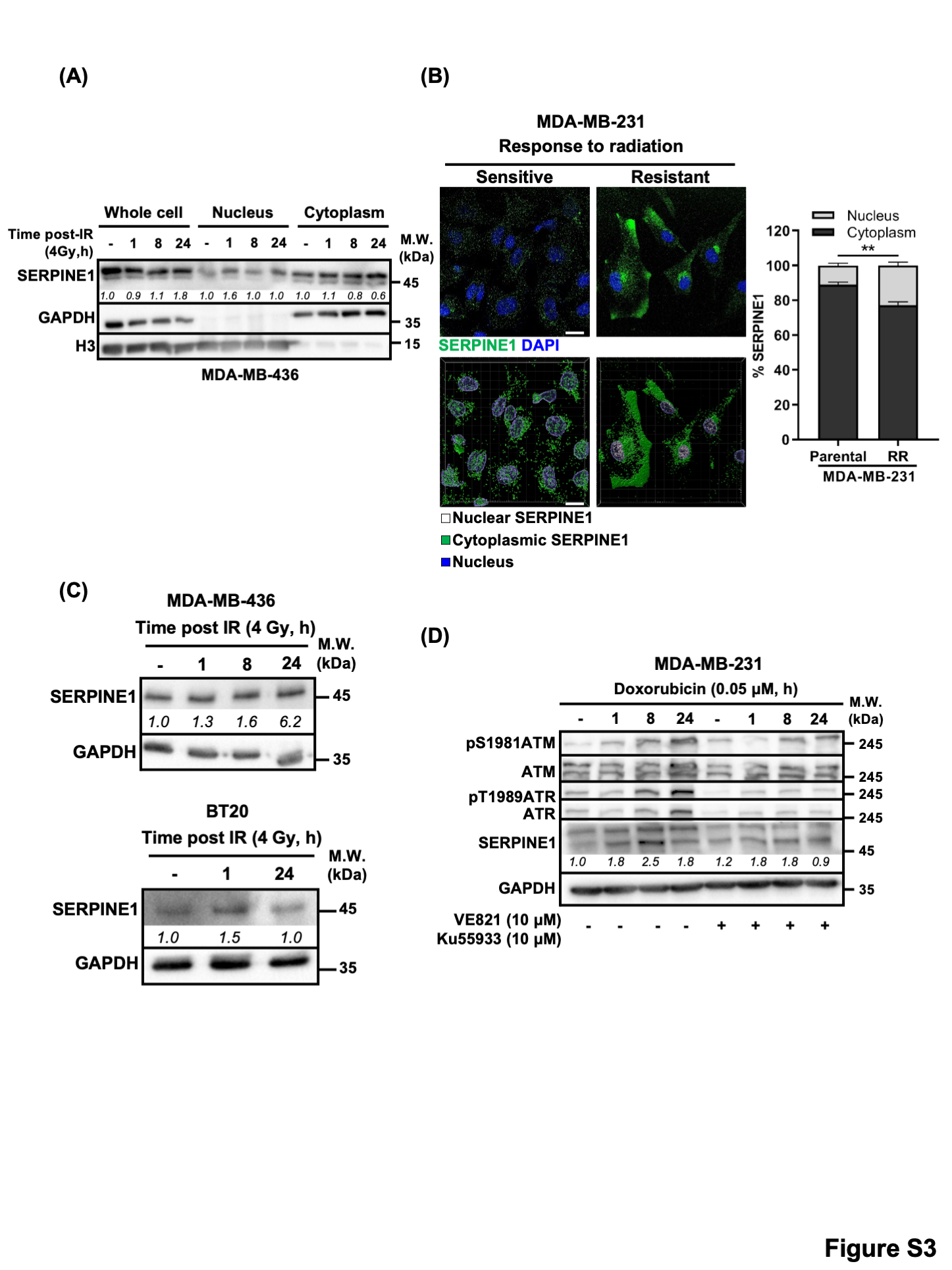

Supplement: Supplementary file 1 — Extended Data [file 41419_2023_5576_MOESM1_ESM.zip › Supplementary data_Su&Kuo_R1/Fig. S3.tiff]

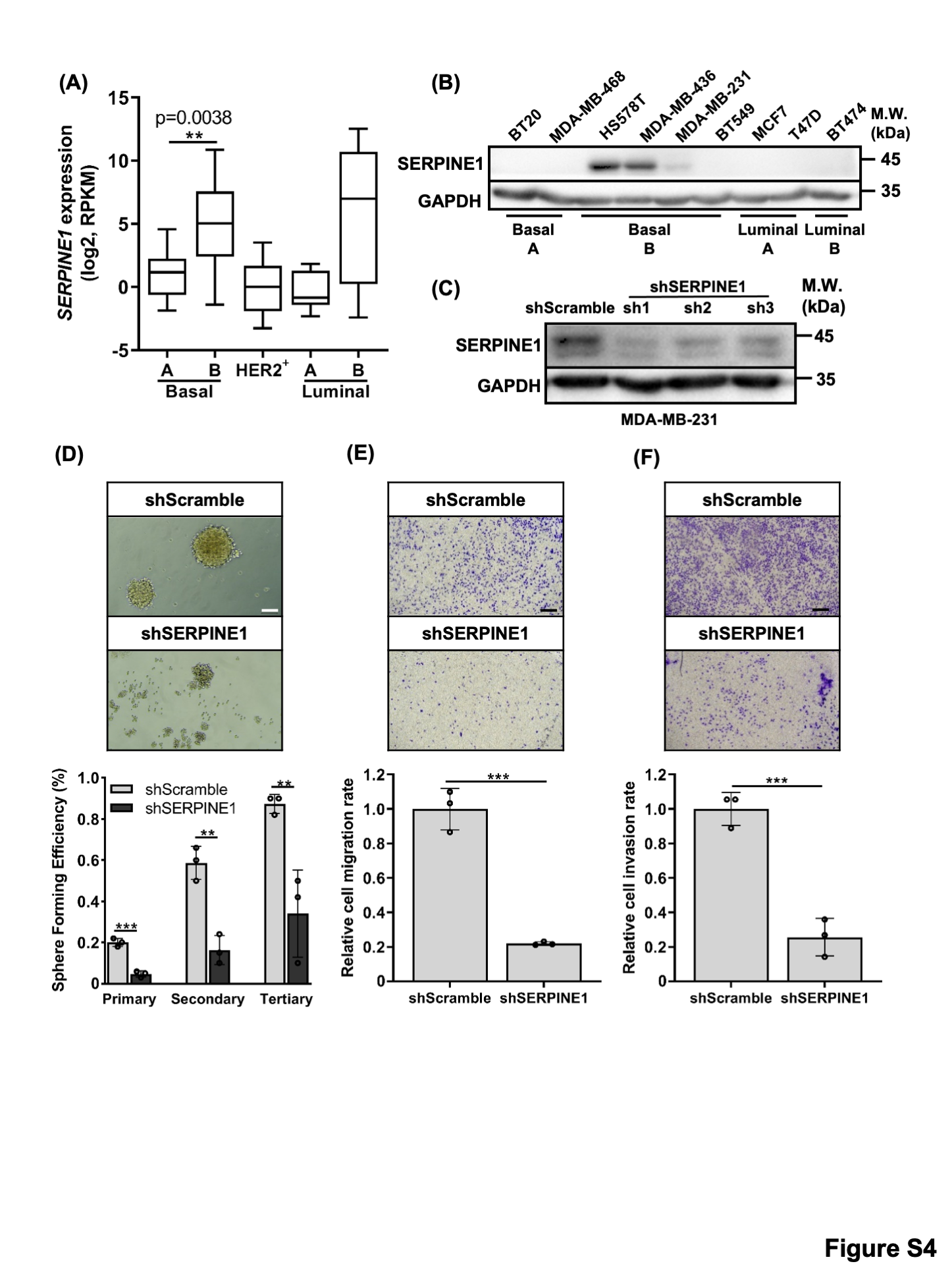

Supplement: Supplementary file 1 — Extended Data [file 41419_2023_5576_MOESM1_ESM.zip › Supplementary data_Su&Kuo_R1/Fig. S4.tiff]

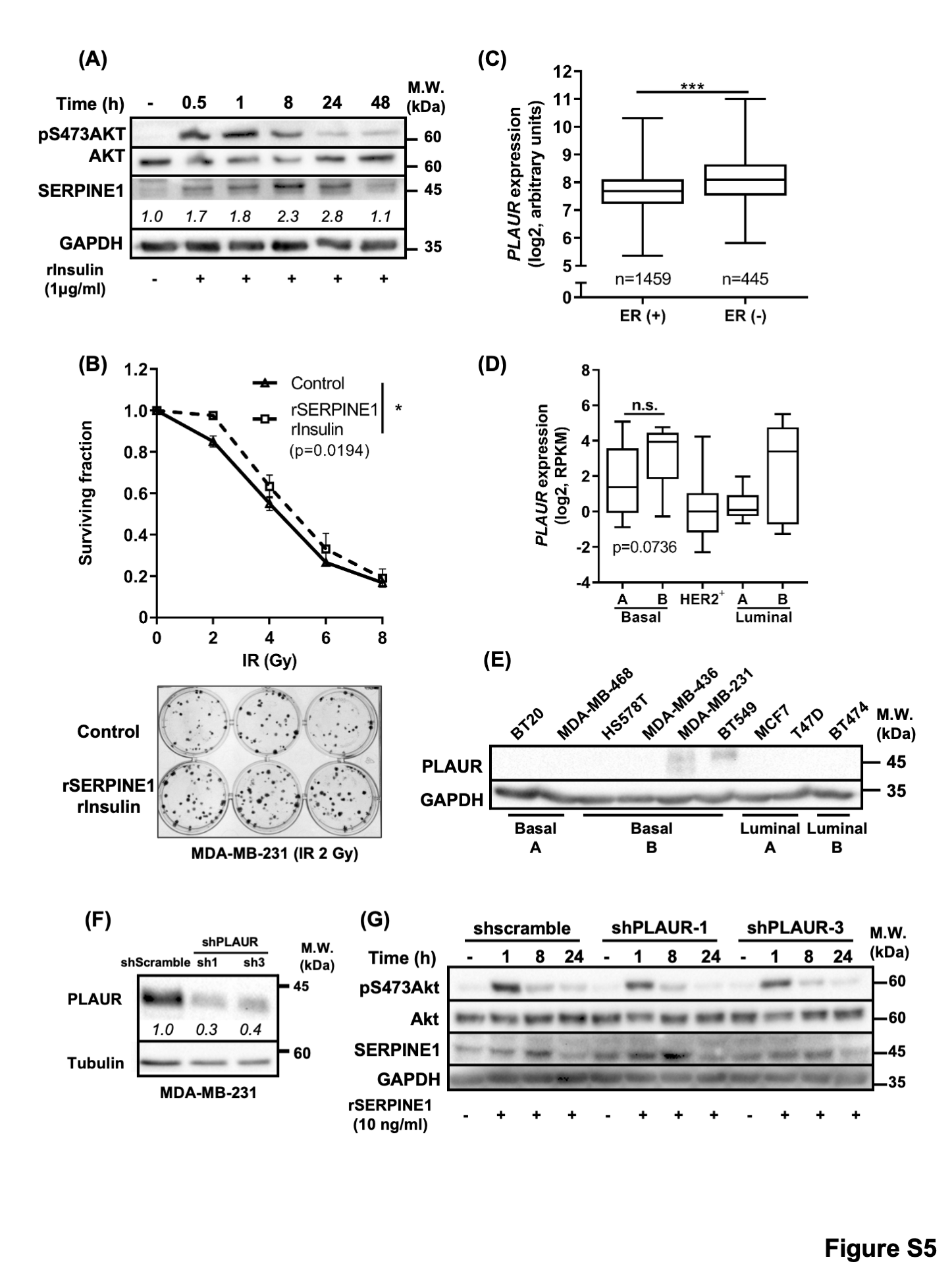

Supplement: Supplementary file 1 — Extended Data [file 41419_2023_5576_MOESM1_ESM.zip › Supplementary data_Su&Kuo_R1/Fig. S5.tiff]

Fig. 1I

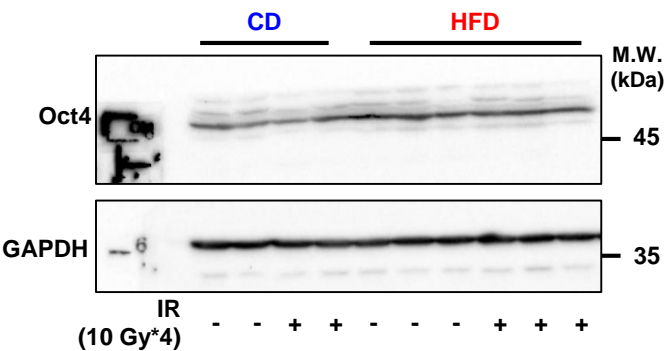

Fig. 3B

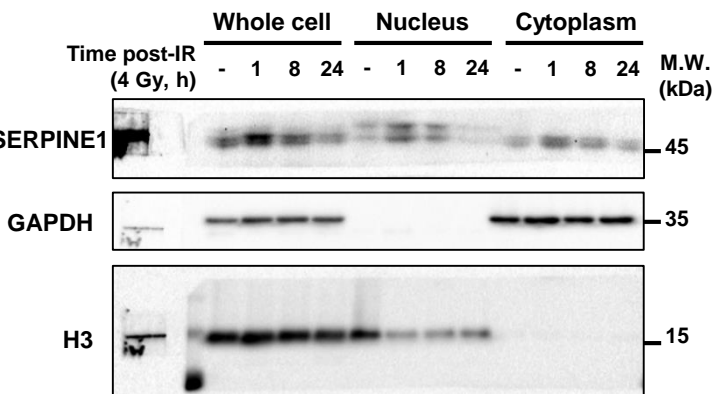

Fig. 3E

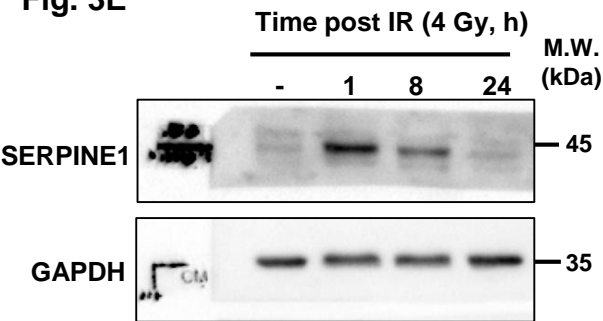

Fig. 3G

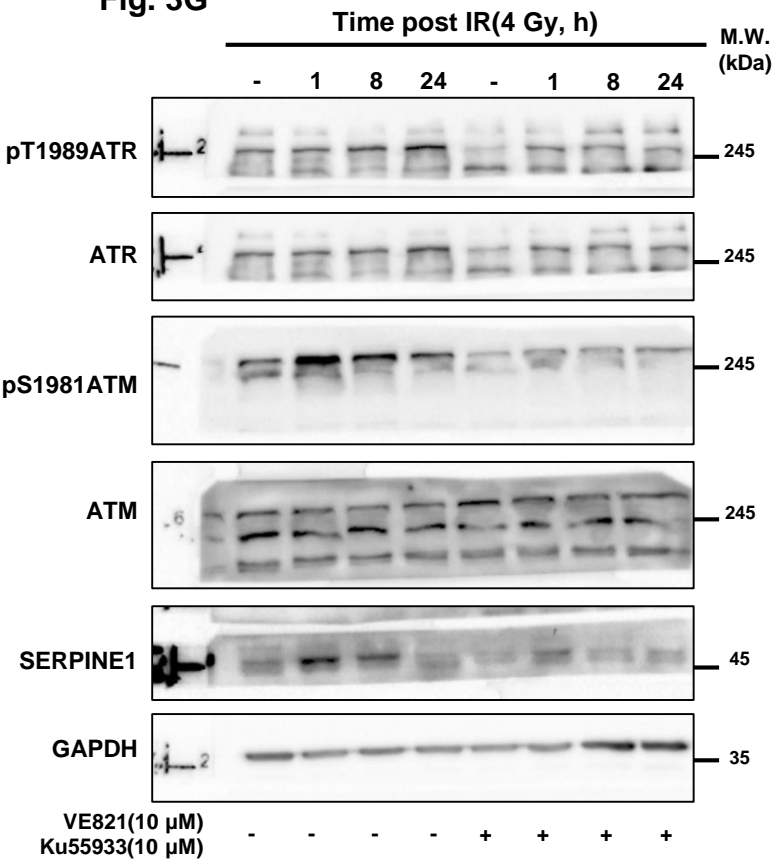

**Fig. 5B**

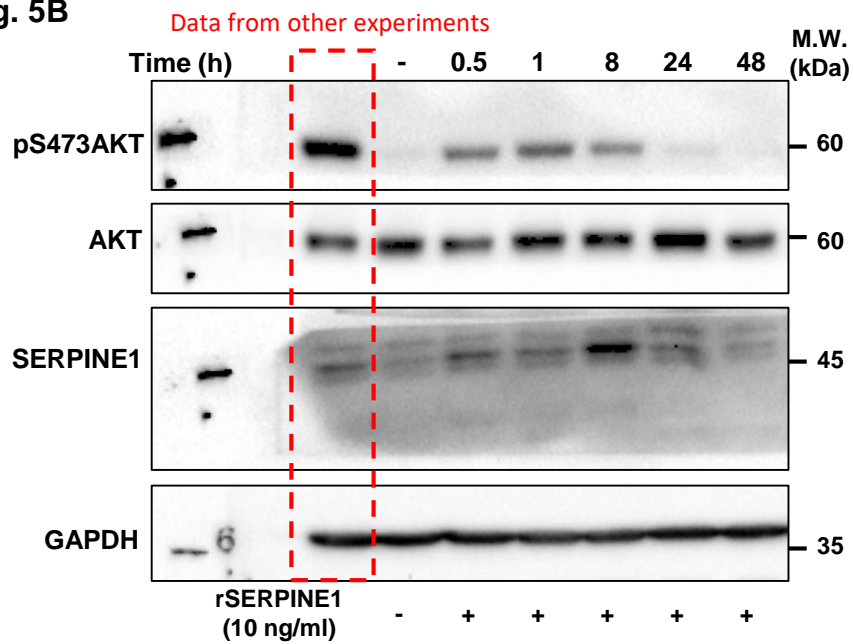

**Fig. 5C**

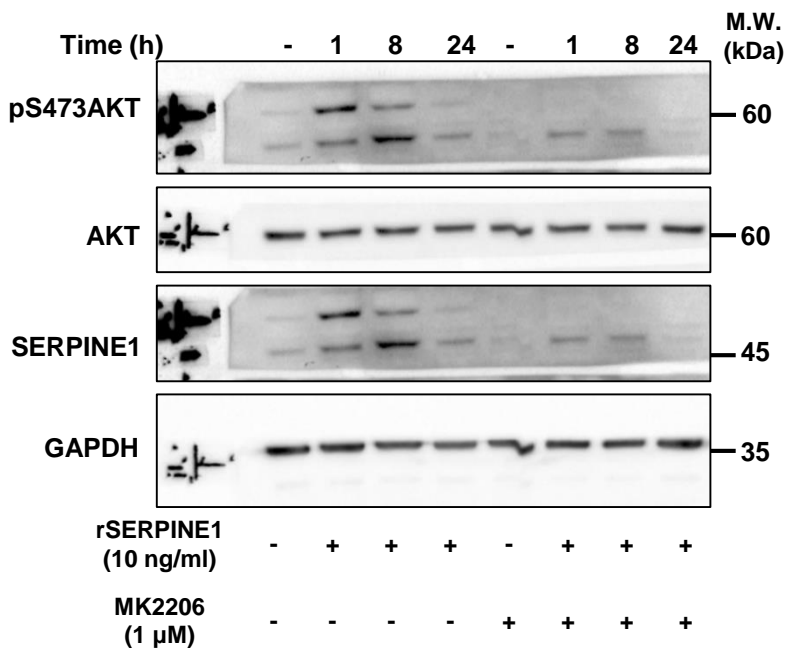

Fig. S2G

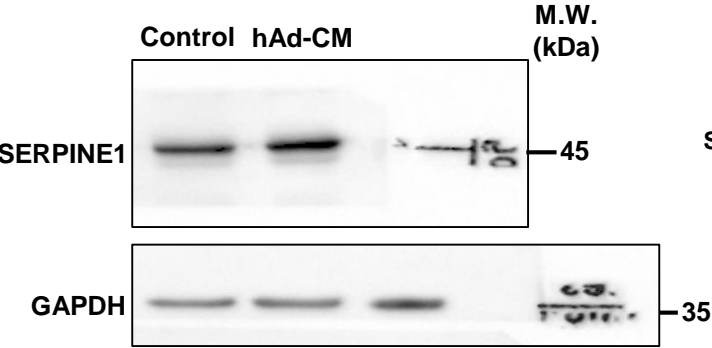

Fig. S3A

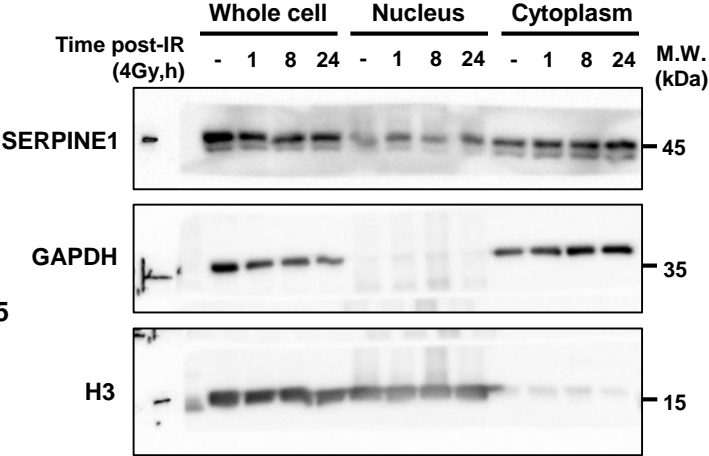

Fig. S3C

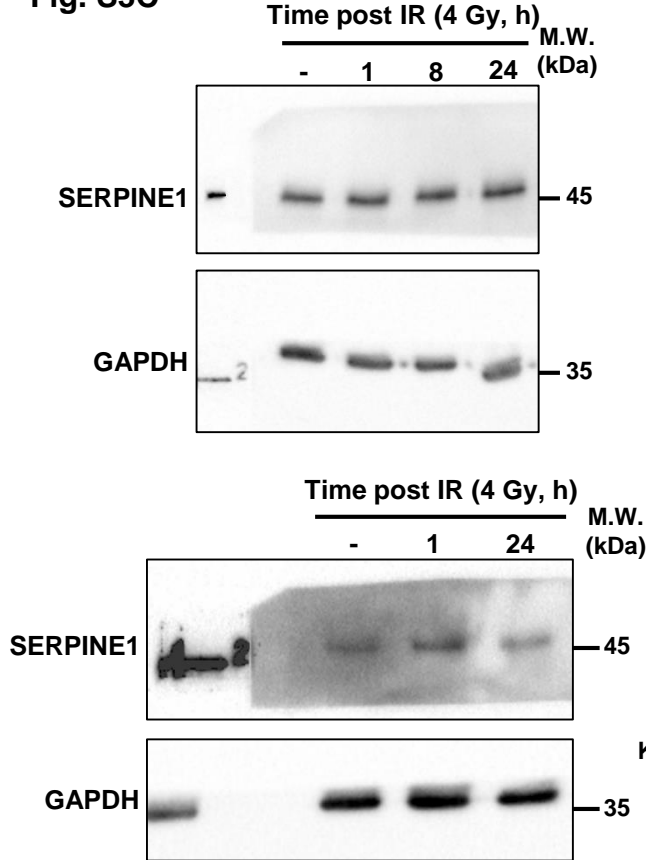

Fig. S3D

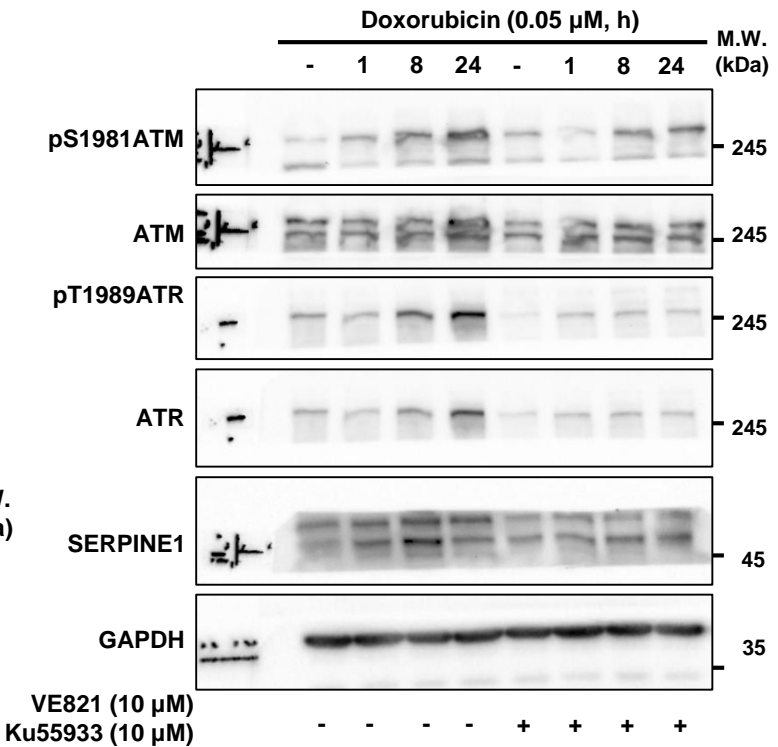

**Fig. S4B**

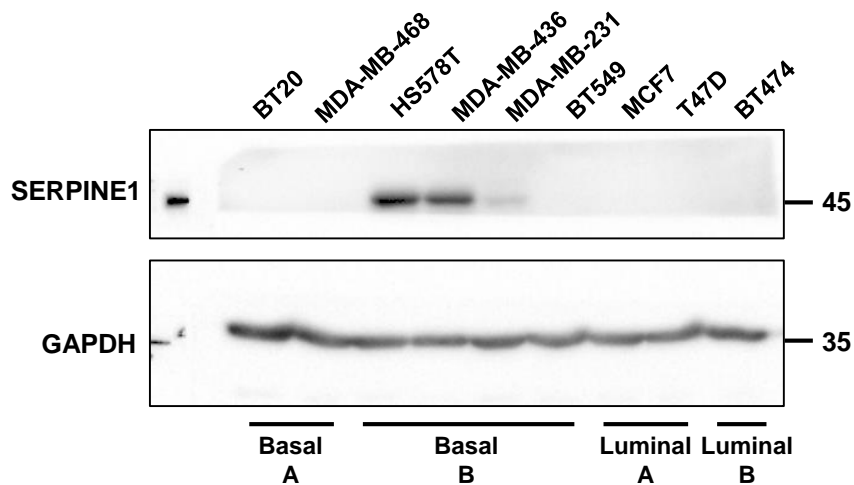

**Fig. S4C**

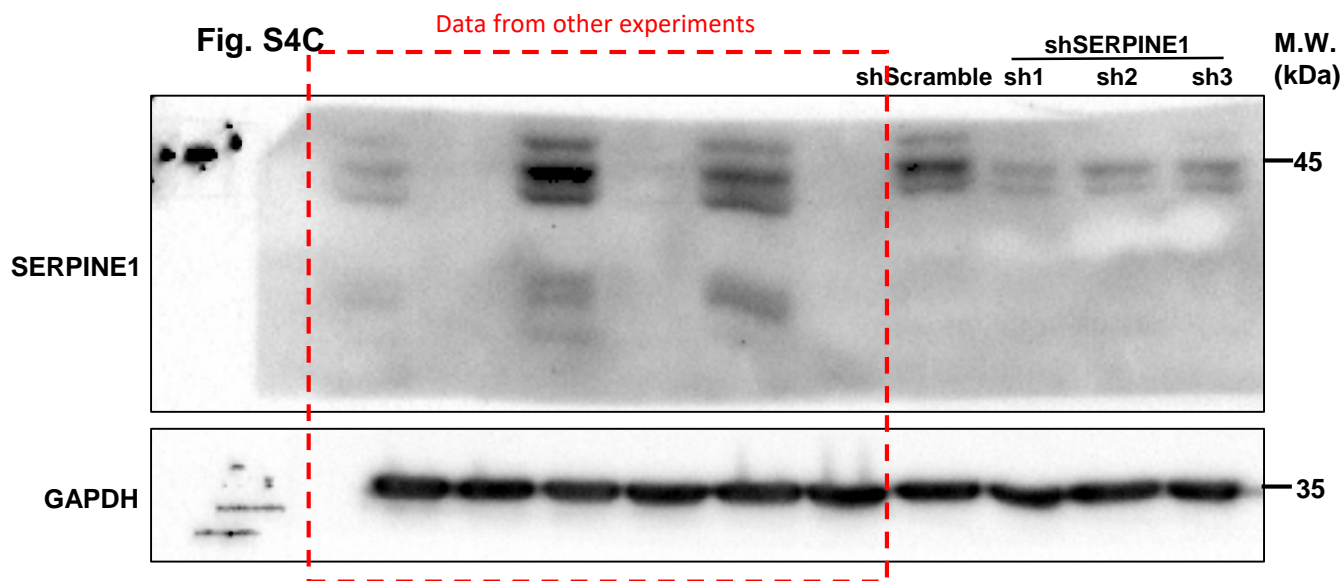

**Fig. S5A**

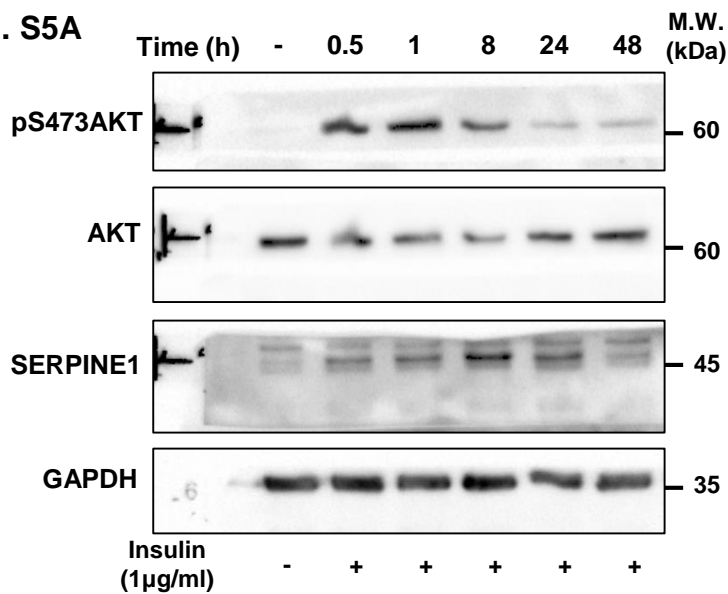

**Fig. S5E**

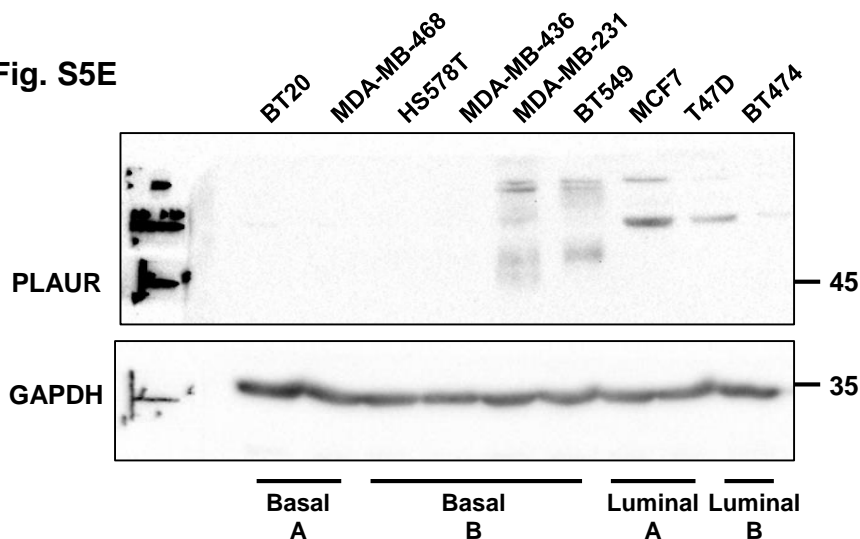

**Fig. S5F**

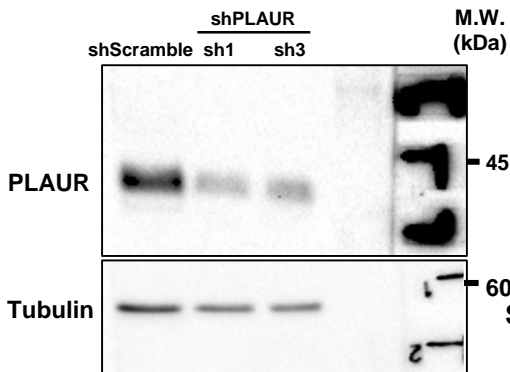

**Fig. S5G**

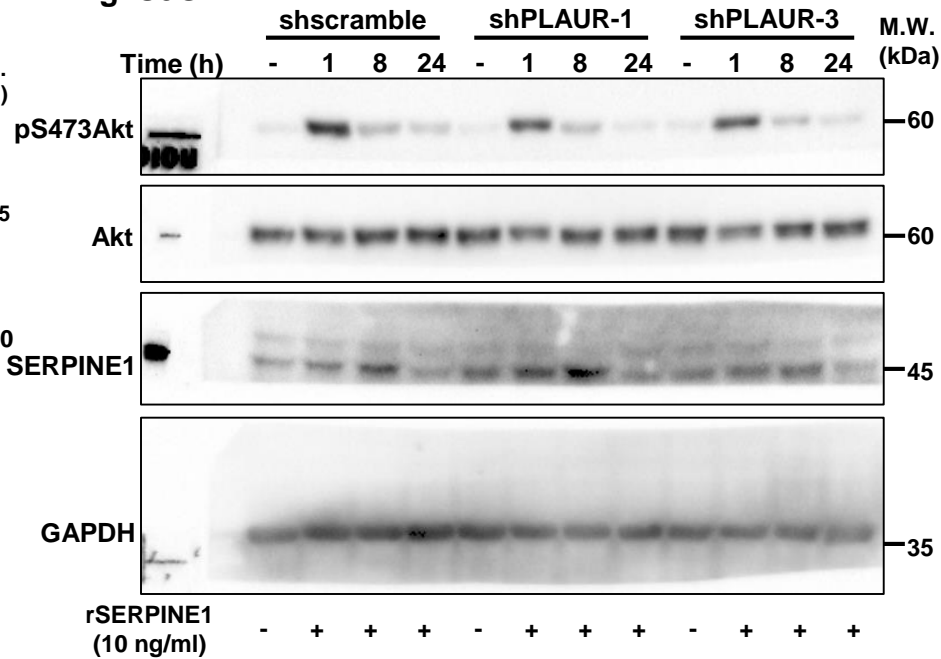

Supplement: Supplementary file 3 — Original Uncropped Western blots [file 41419_2023_5576_MOESM3_ESM.pdf]
